# Proteomic analysis of fipronil-induced molecular defects in spermatozoa

**DOI:** 10.1038/s41598-024-57876-4

**Published:** 2024-04-01

**Authors:** Jeong-Won Bae, Woo-Sung Kwon

**Affiliations:** 1 Department of Animal Science and Biotechnology, Kyungpook National University, Sangju, Republic of Korea; 2 Research Institute for Innovative Animal Science, Kyungpook National University, Sangju, Republic of Korea

**Keywords:** Pesticide-affected spermatozoa, Differentially expressed proteins, Male infertility, Proteomics, Environmental impact, Risk factors

## Abstract

The phenylpyrazole insecticide fipronil has wide-ranging applications from agriculture to public health to control undesirable organisms. However, several studies have reported the residual environmental hazards of fipronil and demonstrated its harmful effects even in mammalian reproduction. Therefore, this study was conducted to demonstrate the mode of action of fipronil on mouse spermatozoa. We treated fipronil to spermatozoa and performed comprehensive function evaluations. Moreover, proteomic analyses were conducted to identify the alteration of protein expression levels in spermatozoa. Most of sperm motility and kinematic parameters and intracellular ATP levels were diminished, and the spontaneous acrosome reaction was promoted after treatment with fipronil. Proteomic analyses revealed altered expression levels of 14 proteins after treatment. These proteins have been reported to be associated with sperm-specific pathways, prominently the cytoskeleton of the sperm, “9 + 2” axoneme composition, metabolism, and fertility. Collectively, our results showed that fipronil alters sperm functional-related proteins and therefore influences male fertility. This study elucidates the possible reproductive toxic hazards associated with male infertility through aberrant suppression of sperm proteins.

## Introduction

Fipronil is a versatile phenylpyrazole insecticide with a wide range of applications such as public health, veterinary medicine, and agriculture. Fipronil disrupts the central nervous system of insects by targeting gamma-aminobutyric acid mediated chloride channel and glutamate-activated chloride channels, and it is more effective against insects than against mammals. Recently, considerable literature has accumulated regarding fipronil residues and its degradants detected in ecosystems such as urban surfaces, streams, groundwater, soil, and indoor dust^1–3^. According to existing research, fipronil residues and its degradants are prevalent in municipal wastewater treatment plants, river sediments, and urban estuarine sediments in USA^4,5^. Furthermore, in Vietnam, the threshold concentration of fipronil reviewed by the US EPA was detected in lake water^6,7^. A particular concern is that fipronil can be exposed and affect daily life such as through groundwater used for public supply^8^. In other words, although fipronil was reported to exhibit selective toxicity to undesirable organisms, the potential risks of human environmental exposure have become an increasing global concern. It is currently well established that fipronil exerts negative effects on various aspects of nontarget organisms, including mammals. A previous study reported that fipronil alters hormone levels and the estrous cycle length; fipronil-affected hormonal imbalance may result in a lower pregnancy rate in rats^9^. Moreover, aggravated ROS levels and DNA damage caused by fipronil increase apoptosis and cell cycle arrest during porcine oocyte maturation^10^. Even in embryos, it was found that fipronil depressed in vitro and in vivo developmental abilities in the mouse^11^. As well, the oxidant activity of fipronil in rats was found to suppress sperm production, reducing the epididymal sperm count^12^. Furthermore, fipronil increases DNA damage and apoptosis of rat spermatozoa, resulting in negative consequences on male fertility^13^. In particular, the previous study reported that fipronil may be involved in capacitation by binding with GABA_A_ receptors, supplanting GABA, which may play a vital role as a putative modulator of sperm^14^. Despite these potential toxicological effects, studies demonstrating the molecular mechanisms of fipronil action on sperm function are still nascent. Therefore, in this study, we performed proteomic profiling of fipronil-treated spermatozoa and then applied bioinformatics to screen proteomic variations and the mechanisms of action of fipronil in mouse spermatozoa.

## Results

### Evaluation of sperm functions and characteristics

**Figure 1:**
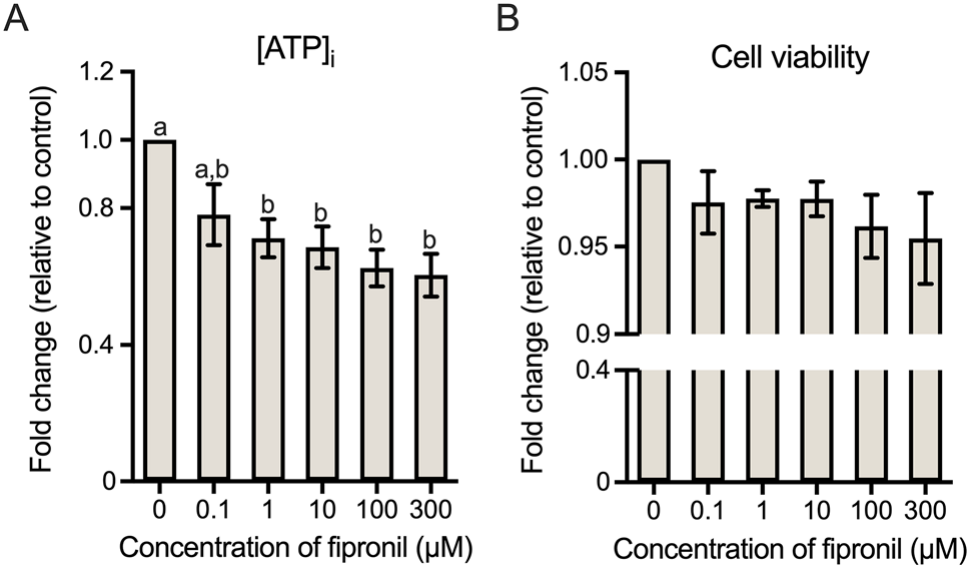
Comparison of intracellular ATP level and cell viability. (**A**) Differences in intracellular ATP levels after treatment with various concentration of fipronil. (**B**) Differences in cell viability between control and fipronil-treated groups. Data represent mean ± SEM, n = 3. Values with different superscripts (^a and b^) indicate significant difference between control and each treatment group (*p* < 0.05).

As shown in Table 1, all parameters of motility, i.e., total sperm motility (MOT) and progressive motility (PR) were significantly decreased with 1 µM fipronil treatment (*p* < 0.05). Among the kinematic parameters, average path velocity (VAP) was significantly decreased at lowest concentration of fipronil treatment (0.1 µM; *p* < 0.05). Curvilinear velocity (VCL) and straight-line velocity (VSL) showed a decreasing trend commencing at 1 µM (*p* < 0.05). In addition, beat cross frequency (BCF) and amplitude of lateral head displacement (ALH) decreased from 10 µM fipronil (*p* < 0.05). There was no significant difference in mean dance (DNM). In the measurements of capacitation status, the percentage of capacitated spermatozoa (B pattern) showed a sharp decline only at the highest concentration of fipronil (300 µM; *p* < 0.05). Meanwhile, the number of acrosome-reacted sperm (AR pattern) were drastically increased from 10 µM fipronil treatment, but there was no significant change in the non-capacitated pattern (F pattern) (*p* < 0.05; Table 1). The intracellular ATP level showed deficiencies at fipronil concentrations of 1, 10, 100, and 300 µM (*p* < 0.05; Fig. 1A). However, there was no statistical difference in cell viability between all treatment groups (Fig. 1B).

**Table 1:** Functional evaluation of fipronil-affected spermatozoa.

Parameters	Concentration (µM)					
	0	0.1	1	10	100	300
Motility						
MOT	82.23 ± 3.50^a^	70.08 ± 4.34^a,b^	64.68 ± 2.51^b^	62.54 ± 2.36^b^	62.29 ± 1.26^b^	56.68 ± 4.79^b^
PR	77.38 ± 3.33^a^	65.43 ± 4.37^a,b^	59.40 ± 2.64^b^	57.10 ± 2.15^b^	56.84 ± 1.41^b^	53.05 ± 4.39^b^
Kinematics						
VCL	113.00 ± 5.56^a^	90.35 ± 9.67^a,b^	84.22 ± 5.11^b^	79.63 ± 3.93^b^	75.27 ± 2.71^b^	71.00 ± 5.50^b^
VSL	39.14 ± 2.97^a^	26.20 ± 5.24^a,b^	21.49 ± 3.20^b^	20.30 ± 2.33^b^	20.89 ± 2.21^b^	21.09 ± 1.28^b^
VAP	60.18 ± 3.76^a^	42.81 ± 5.86^b^	37.59 ± 3.52^b^	34.58 ± 1.99^b^	34.48 ± 1.51^b^	32.59 ± 2.78^b^
BCF	7.18 ± 0.44^a^	5.94 ± 0.75^a,b^	5.58 ± 0.51^a,b^	4.88 ± 0.37^b^	4.70 ± 0.10^b^	4.59 ± 0.12^b^
DNM	16.57 ± 1.06	19.36 ± 1.67	21.93 ± 2.62	21.25 ± 2.30	19.58 ± 2.50	17.15 ± 0.99
ALH	4.57 ± 0.21^a^	3.86 ± 0.35^a,b^	3.60 ± 0.18^a,b^	3.38 ± 0.11^b^	3.29 ± 0.11^b^	2.98 ± 0.24^b^
Capacitation status						
AR	16.61 ± 0.98^a^	15.60 ± 3.32^a,b^	18.61 ± 7.40^a,b^	30.05 ± 11.29^b^	55.46 ± 15.87^b^	65.49 ± 4.90^b^
B	47.81 ± 3.88^a^	40.55 ± 5.30^a,b^	40.63 ± 6.33^a,b^	39.86 ± 8.17^a,b^	24.01 ± 7.22^a,b^	16.37 ± 3.62^b^
F	35.59 ± 4.36	43.85 ± 3.40	40.76 ± 2.96	30.09 ± 6.19	20.53 ± 9.54	18.14 ± 6.62

Sperm motility and kinematic values are presented as mean ± SEM (n = 4). MOT = Total sperm motility (%); PR = Progressive motility (%); VCL = Curvilinear velocity (μm/s); VSL = Straight-line velocity (μm/s); VAP = Average path velocity (μm/s); BCF = Beat cross frequency (Hz); DNM = Mean dance (μm); ALH = Mean amplitude of head lateral displacement (μm); AR = Acrosome-reacted pattern (%); B = capacitated pattern (%); F = non-capacitated pattern (%). Values with different superscript letters ^(a and b)^ indicate significant difference between control and each treatment group (*p* < 0.05).

### Identification of differentially expressed proteins

**Figure 2:**
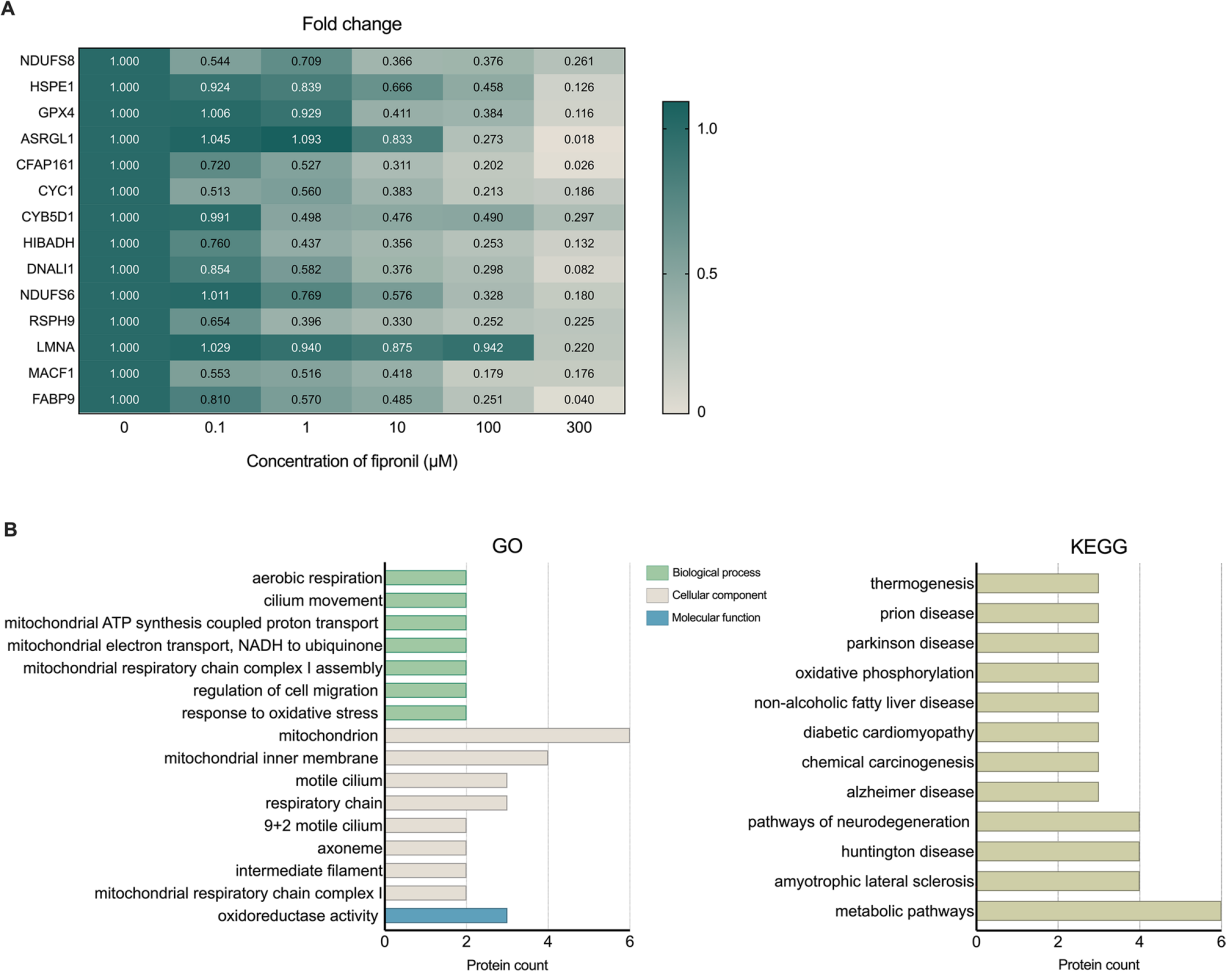
Differentially expressed proteins (DEPs) and related pathways were measured and visualized. (**A**) Heat maps visualized the expression level of 14 DEPs after treatment with each concentration of fipronil. (**B**) GO and KEGG depict the discovered related pathways and protein counts in histogram.

The measurement and identification of proteins and differential expression levels were accomplished through 2-DE, LC–MS/MS, and MASCOT database search. As shown in Table 2 and Supplementary Table S1, the proteomic analysis identified 14 differentially expressed proteins (DEPs) that were significantly downregulated (> threefold) due to fipronil treatment (*p* < 0.05). The normalized expression values of each DEP are shown in Fig. 2A.

**Table 2:** Differentially expressed (> threefold) proteins identified by LC–MS/MS and MASCOT database searching.

Accession	Symbol	Description	Score*	MW
Q8K3J1	NDUFS8	NADH dehydrogenase [ubiquinone] iron-sulfur protein 8, mitochondrial	1256	24,479
Q64433	HSPE1	10 kDa heat shock protein, mitochondrial	1035	10,956
O70325-3	GPX4	Isoform Nuclear of Phospholipid hydroperoxide glutathione peroxidase	903	29,803
Q8C0M9	ASRGL1	Isoaspartyl peptidase/L-asparaginase	722	34,385
Q6P8Y0	CFAP161	Cilia- and flagella-associated protein 161	571	34,651
Q9D0M3	CYC1	Cytochrome c1, heme protein, mitochondrial	528	35,533
Q5NCY3	CYB5D1	Cytochrome b5 domain-containing protein 1	416	26,759
Q99L13	HIBADH	3-hydroxyisobutyrate dehydrogenase, mitochondrial	402	35,816
Q8BVN8	DNALI1	Axonemal dynein light intermediate polypeptide 1	286	29,947
P52503	NDUFS6	NADH dehydrogenase [ubiquinone] iron-sulfur protein 6, mitochondrial	282	13,183
Q9D9V4	RSPH9	Radial spoke head protein 9	109	31,331
P48678	LMNA	Prelamin-A/C	90	74,238
Q9QXZ0	MACF1	Microtubule-actin cross-linking factor 1	73	831,878
O08716	FABP9	Fatty acid-binding protein 9	72	15,017

*MASCOT score is − 10 log (P), where *p* is the probability that the observed match is a random event. Protein scores > 55 indicate identity or extensive homology (*p* < 0.05).

### Verification of DEPs

**Figure 3:**
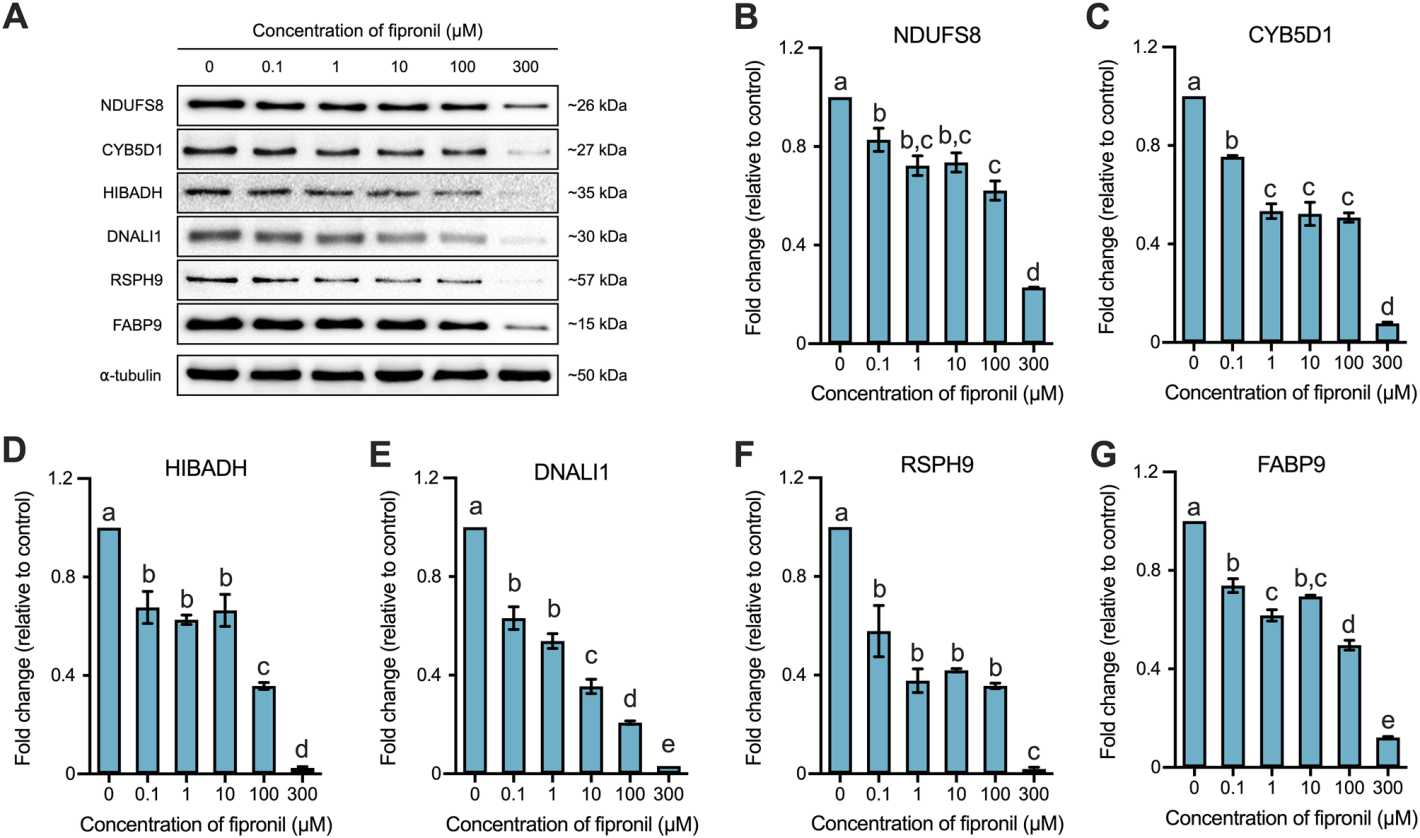
The histogram of fold change of the representative proteins relative to control. The expression ratios of representative 6 DEPs are depicted in the histogram. (**A**) NDUFS8, CYB5D1, HIBADH, DNALI1, RSPH9, and FABP9 were detected approximately at 26, 27, 35, 30, 57, and 15 kDa, respectively. (**B**) The expression level of NDUFS8. (**C**) The expression level of CYB5D1. (**D**) The expression level of HIBADH. (**E**) The expression level of DNALI1. (**F**) The expression level of RSPH9. (**G**) The expression level of FABP9. Data represent mean ± SEM, n = 3. Values with different superscripts (^a, b, c, d, and e^) indicate significant difference between control and each treatment group (*p* < 0.05).

The verification of DEPs was performed by western blotting and illustrated in Fig. 3. Among the 14 DEPs, we confirmed that the expression of cytochrome b5 domain-containing protein 1 (CYB5D1), axonemal dynein light intermediate polypeptide 1 (DNALI1), fatty acid-binding protein 9 (FABP9), 3-hydroxyisobutyrate dehydrogenase, mitochondrial (HIBADH), NADH dehydrogenase [ubiquinone] iron-sulfur protein 8, mitochondrial (NDUFS8), and radial spoke head protein 9 homolog (RSPH9) significantly decreased in all treatment groups. Moreover, the expression level of all six proteins were drastically decreased at the highest fipronil concentration (300 µM) relative to the control (*p* < 0.05; Fig. 3).

### Bioinformatics analysis of DEPs

The DEPs were analyzed by the DAVID database using GO and KEGG analyses. The seven biological processes, eight cellular components, and one molecular function related pathways were categorized (*p* < 0.05; Table 3 and Fig. 2B). In addition, KEGG enrichment analysis confirmed 12 related pathways, including metabolic pathways and oxidative phosphorylation process (*p* < 0.05; Table 3 and Fig. 2B).

**Table 3:** Pathway related proteins and count.

Category	Count	Symbol
Biological process		
Aerobic respiration	2	NDUFS6, NDUFS8
Cilium movement	2	DNALI1, RSPH9
Mitochondrial ATP synthesis coupled proton transport	2	NDUFS6, NDUFS8
Mitochondrial electron transport, NADH to ubiquinone	2	NDUFS6, NDUFS8
Mitochondrial respiratory chain complex I assembly	2	NDUFS6, NDUFS8
Regulation of cell migration	2	LMNA, MACF1
Response to oxidative stress	2	GPX4, NDUFS8
Cellular component		
Mitochondrion	6	CYC1, GPX4, HIBADH, HSPE1, NDUFS6, NDUFS8
Mitochondrial inner membrane	4	CYC1, GPX4, NDUFS6, NDUFS8
Motile cilium	3	CFAP161, DNALI1, RSPH9
Respiratory chain	3	CYC1, NDUFS6, NDUFS8
9 + 2 motile cilium	2	DNALI1, RSPH9
Axoneme	2	DNALI1, RSPH9
Intermediate filament	2	LMNA, MACF1
Mitochondrial respiratory chain complex I	2	NDUFS6, NDUFS8
Molecular function		
Oxidoreductase activity	3	GPX4, HIBADH, NDUFS8
KEGG Pathway		
Metabolic pathways	6	ASRGL1, CYC1, GPX4, HIBADH, NDUFS6, NDUFS8
Amyotrophic lateral sclerosis	4	CYC1, DNALI1, NDUFS6, NDUFS8
Huntington disease	4	CYC1, DNALI1, NDUFS6, NDUFS8
Pathways of neurodegeneration	4	CYC1, DNALI1, NDUFS6, NDUFS8
Alzheimer disease	3	CYC1, NDUFS6, NDUFS8
Chemical carcinogenesis	3	CYC1, NDUFS6, NDUFS8
Diabetic cardiomyopathy	3	CYC1, NDUFS6, NDUFS8
Non-alcoholic fatty liver disease	3	CYC1, NDUFS6, NDUFS8
Oxidative phosphorylation	3	CYC1, NDUFS6, NDUFS8
Parkinson disease	3	CYC1, NDUFS6, NDUFS8
Prion disease	3	CYC1, NDUFS6, NDUFS8
Thermogenesis	3	CYC1, NDUFS6, NDUFS8

### Construction of PPI network and signaling pathway

**Figure 4:**
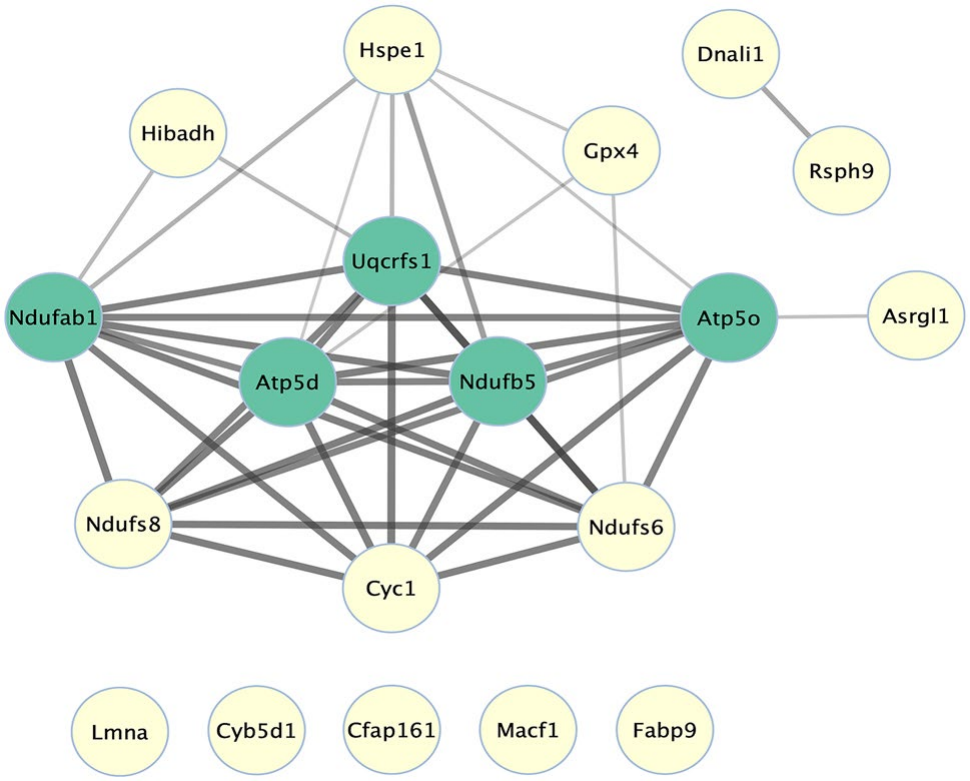
Construction of protein–protein interaction (PPI) network between DEPs. The schematic of PPI network among the 14 DEPs in *Mus musculus* is described using STRING. The 14 DEPs are circled in lemon and the 5 proteins in blue-green circles evidence new proteins that are functionally associated with 14 DEPs. A confidence cutoff for interaction was set to medium (0.400).

**Figure 5:**
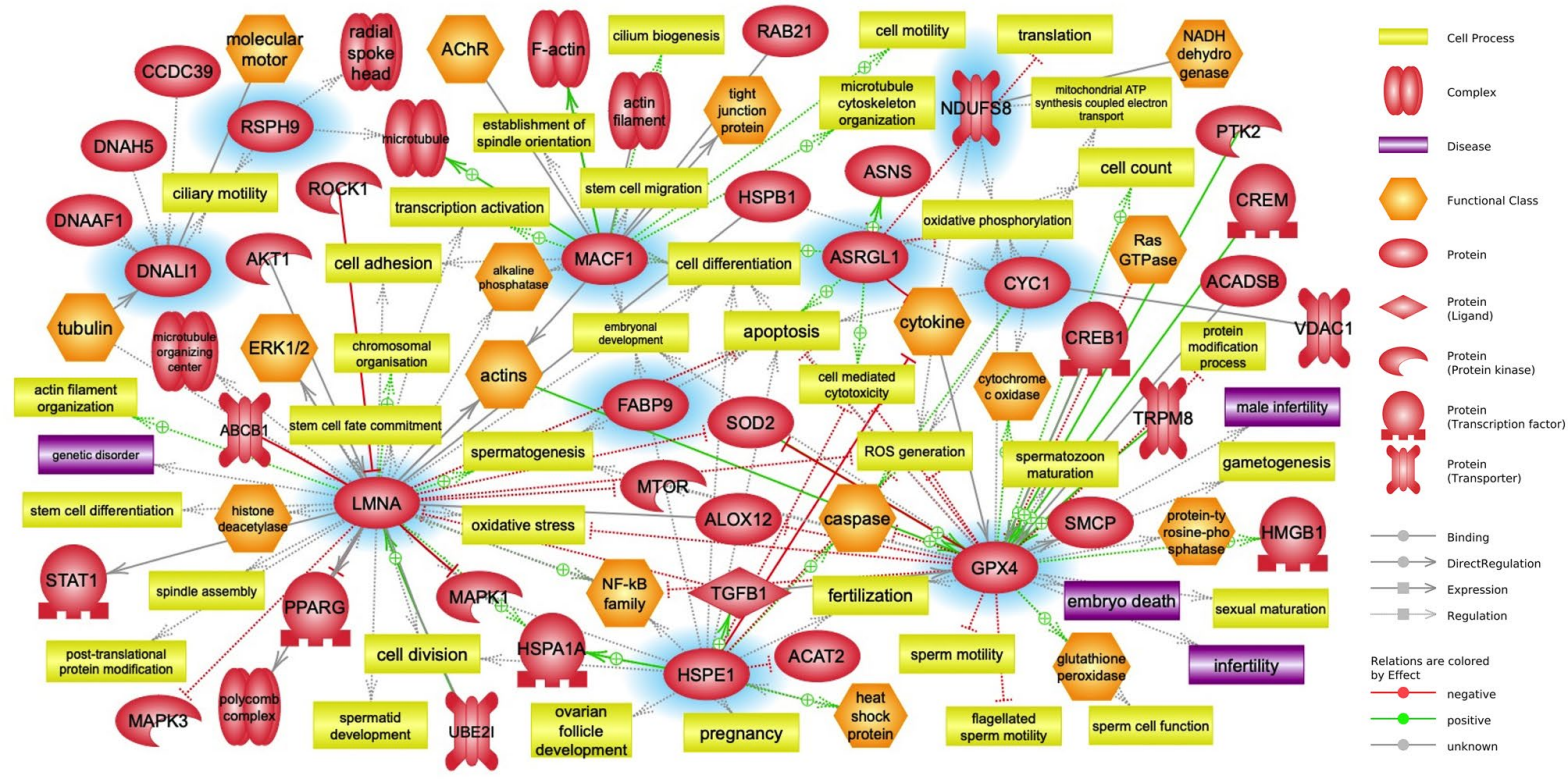
Construction of signaling pathway between DEPs. The signaling pathway of DEPs is illustrated using Pathway Studio. The DEPs are highlighted in blue background.

The PPI network was constructed using Cytoscape with the STRING network. The results of the interaction analysis are summarized in Fig. 4. A total of 19 nodes and 40 edges were identified in *Mus musculus*. HIBADH, 10 kDa heat shock protein, mitochondrial (HSPE1), Isoform Nuclear of Phospholipid hydroperoxide glutathione peroxidase (GPX4), DNALI1, RSPH9, Isoaspartyl peptidase/L-asparaginase (ASRGL1), NDUFS8, Cytochrome c1, heme protein, mitochondrial (CYC1), and NADH dehydrogenase [ubiquinone] iron-sulfur protein 6, mitochondrial (NDUFS6) were found to interact with each other. The related signaling pathway of 10 proteins [RSPH9, DNALI1, Prelamin-A/C (LMNA), Microtubule-actin cross-linking factor 1 (MACF1), FABP9, HSPE1, ASRGL1, NDUFS8, CYC1, and GPX4] was constructed. All 10 DEPs interact with various proteins and are significantly related to cell processes such as sperm motility, reproductive cell development and maturation, and fertilization. Especially, it has been found that proteins LMNA and GPX4 are associated with diseases such as genetic disorder, embryo death, and infertility (*p* < 0.05; Fig. 5).

## Discussion

Fipronil is a widespread insecticidal ingredient that interrupts GABA and GluCl channels, impairing the central nervous system of the target organism. Although fipronil may be less toxic to mammals due to the absence of GluCl channels, recent studies have consistently shown that exposure to fipronil is associated with adverse effects in mammals^9–13^. Nevertheless, there is still scarce information regarding the potential impact of fipronil on sperm functions and the underlying molecular mechanisms. To explore the mechanism of fipronil action on the sperm proteome, we first evaluated the comprehensive sperm function.

Evaluation of sperm motility and kinematics revealed that fipronil treatment overall declined the sperm motility and kinematics parameters. Fipronil-treated spermatozoa exhibited decreased total motility, progressive motility, VCL, VSL, VAP, BCF, and ALH, although the total motility value was higher than the lower reference limit proposed by the WHO and thus estimated as normal motility values (Table 1). Similar to our results, a previous study demonstrated that perinatal exposure to fipronil reduced sperm motility and increased the number of nonmobile sperm in male rats^15^. Bae and Kwon^14^ also showed that fipronil suppressed the overall sperm motility and kinematics. Therefore, fipronil may affect sperm motility and kinematics. The highly motile specialized organelle flagella are components of the sperm tail and enable the sperm to produce locomotion. Frequently, axoneme deficiencies in sperm cause poor motility and results in infertility. The flagella of sperm are primarily organized with microtubules (MTs) composed of “9 + 2” axonemal complexes. The axonemal complexes consist of nine outer doublet microtubules, and two central pair interacts with and regulates ciliary movement. In the interaction, T-shaped radial spoke complexes with outer and inner dynein arms (ODA and IDA) assembling nine MTs with a central singlet. In this structure, sperm flagellar motility was coordinated by numerous dyneins in the axoneme using ATP^16^. In the present study, the expression levels of four DEPs, CFAP161, CYB5D1, DNALI1, and RSPH9 were significantly decreased, and CYB5D1, DNALI1, and RSPH9 positively correlated with sperm motility and kinematics, except DNM (Table 2, Supplementary Table S2, and Fig. 2A and 3). CYB5D1 is an RS component and coordinates the beating of flagella. A recent study showed that CYB5D1 mutation and knockdown zebrafish impaired coordinated ciliary beating in the olfactory epithelium and otic vesicle^17^. The component of dynein molecular motors, DNALI1, was previously reported to be expressed in testes, spermatids, and spermatocytes as well as the flagella of spermatozoa supporting axonemal dynein^18,19^. DNALI1 is localized along the IDA, which regulates flagellar beating, and the function of DNALI1 in the flagella of spermatozoa is still nascent, suggesting that it is involved in the generation of sperm beating^20,21^. In the DNALI1 knockdown zebrafish, ciliopathy phenotypes and depleted ODA has been observed, and revealed to be related to primary ciliary dyskinesia (PCD)^22^. PCD is a multisystemic cilia or flagella disorder of cells, including sperm, and closely related to male infertility. It is also known that RSPH9, which was differently expressed in our study, is linked to PCD, and a mutation of RSPH9 in patients resulted in RS abnormalities^23^. Previous studies have reported that in the “9 + 2” axonemal complex structure, the specific protein RSPH9 is a component of the axoneme and required for RS assembly and modulates the movement of axoneme in *Chlamydomonas,* mice, and humans^24,25^. Although the precise mechanism of Cilia- and flagella-associated protein 161 (CFAP161) remains nascent, CFAP161 was detected in testes^26^. A recent study reported that CFAP161 localized at the axoneme and was observed in the late stages of spermiogenesis and in spermatozoa. Hence, CFAP161-knockdown zebrafish lost ODAs, reducing the beating frequency of flagella^27^. Through this study, it was possible to confirm that the expression of CFAP161 was inhibited by fipronil. Synthetically, evidence shows that fipronil can alter sperm motility-related proteins. Consistent with this notion, the result of decreasing sperm motility and kinematics may be influenced by the diminished motility and the decreased expression of flagellum structure-related proteins induced by fipronil.

As a result of the capacitation status, during the capacitation process pattern was significantly decreased, and the non-capacitated spermatozoa tended to decrease, but no significant results were derived. Especially, the number of acrosome-reacted spermatozoa was anomalously increased, and according to the results of the present study, an approximately four-fold change was measured at the highest fipronil concentration (300 µM) relative to the control (Table 1). In previous research, the acrosome reaction was initiated by GABA in human spermatozoa^28^, and another earlier study has also demonstrated that fipronil binds with GABA_A_ receptor instead of GABA^1^. Based on our study, we infer that spontaneous acrosome reaction was increased by fipronil. Furthermore, of the 14 identified DEPs, the expression of FABP9, HSPE1, and LMNA was decreased and related to sperm head. FABP9 is a testis- and germ cell-specific fatty acid-binding protein expressed during postnatal testicular development^29,30^ and identified as a perinuclear theca enriched in proteins in sperm^31^. Accordingly, Selvaraj et al.^29^ reported the presence of FABP9 in the post acrosomal region of the perinuclear theca, and *Fabp9* knockout mice exhibited severe morphological abnormality primarily in the sperm head, even including fertile ones, and there was no significant change in litter size. Our western blot analysis revealed a significant decrease in the expression level of FABP9 in the 0.1 µM treatment group and a sharp decline in the 300 µM treatment group (Fig. 3G). HSPE1, also known as heat shock protein 10, has been expressed in the testes and epididymis of mouse spermatozoa^32,33^. Previously, HSPE1 was observed in the surface of sperm and predicted to play a role in capacitation, and after capacitation, the expression level of sperm surface HSPE1 was drastically increased^33^. However, as shown in Fig. 2A, the expression of HSPE1 significantly decreased despite an increase in the acrosome reaction. Prelamin-A/C is a component of the nuclear lamina; Shen et al.^34^ reported that LMNA plays an essential role in spermatogenesis for the structural and functional development of sperm head and acrosome. Moreover, spermatogenesis was found to be disrupted accompanied by increased apoptosis in *Lmna*^–/–^ mice^35^. Based on these data, we infer that the increase in acrosome reaction indicates that fipronil-induced a spontaneously abnormal complex reaction of the acrosome. On this basis, it is speculated that the fipronil-induced silencing of sperm head-related DEPs affected the abnormally induced acrosome reaction (Table 1 and Fig. 3G). Therefore, we suggest a further study with more focus on the increased spontaneous acrosome reaction.

During the various phases of differentiation, the spermatozoa of individual taxa have a conformational pattern optimized for the penetration of the zona pellucida. During differentiation, a deficiency of the morphological perspective of sperm encompasses adverse outcomes on male fertility. From the structural point of view, numerous proteins compose the spermatozoa, and their complementary cooperation enables the sperm cell to maintain and generate the structure and movement biomechanically. Of the 14 identified DEPs, MACF1 has been known to be primarily involved in cytoskeleton (Table 2, Fig. 2A and 3). Previously, MACF1 was known as an important cytoskeletal crosslinking protein that binds actin filaments to microtubules^36,37^. Furthermore, another previous reported that deletion of *Macf1* attenuates cytoskeletal arrangement^38^, and knockdown of *Macf1* results in less stable F-actin and microtubule arrangement by the RhoA/ROCK1 signaling pathway in mice^39^. MACF1 mRNA was expressed in the mouse embryo^37^, and it is also involved in embryonic development based on the finding that *Macf1*^*−/−*^ embryos died before birth^40^. Corroborating the previous findings, our results also elucidated that MACF1 binds with actin filament and is related to embryonic development (Fig. 5).

We discovered that, despite exhibiting a dose-dependently diminished tendency, the intracellular ATP level significantly decreased and reached a plateau of decline at 1 µM fipronil treatment (Fig. 1A). Of the 14 DEPs, the proteins CYC1, NDUFS6, NDUFS8 were related to sperm metabolism (Table 2, Fig. 2A, 3, and 5). ASRGL1 is an enzyme that catalyzes the hydrolysis of l-asparagine that was previously found to be expressed during oocyte development^41^ and in the sperm tail of rats and humans. Hashemitabar et al.^42^ also found that ASRGL1 was highly expressed in cases of asthenozoospermia. However, the gene expression in asthenozoospermia cases was downregulated compared with that in normozoospermia cases^43^. In our study, the protein expression level was downregulated after treatment with fipronil and positively correlated with sperm motility (Table 1, Supplementary Table S2, and Fig. 2A). CYC1 is a subunit of mitochondrial complex III of the mitochondrial electron transport chain, which is linked to oxidative phosphorylation and has been reported to be associated with sperm ROS production^44,45^. Downregulation of CYC1 reduces ATP production, whereas AMP production increases. Thus, knockdown of CYC1 sharply decreased the activation of mitochondrial complex III and activated AMPK in human breast cancer cells^46^. Moreover, an abnormal processing of CYC1 increased ROS production accompanied by affecting fertility in mice^44^. Therefore, it is plausible that diminished expression of CYC1 may related with deficiencies in ATP production (Fig. 1A). HIBADH plays a major role in catabolism, and a previous study showed that HIBADH, expressed during spermiogenesis in humans, is localized in the head and mid-piece of mature human spermatozoa, and diminished expression of HIBADH was related to low sperm motility^47^. Furthermore, HIBADH was expressed in the mid-piece of bull sperm and can be used as a motility-related marker^48^. Our results support the evidence from previous observations. In our study, the expression level of HIBADH positively correlated with sperm motility and intracellular ATP levels (Supplementary Table S2). NDUFS6 and NDUFS8 are subunits of the NADH dehydrogenase (ubiquinone) complex I that is involved in the respiratory electron transport chain. In *Ndufs6*^−/−^ mice, bone marrow-derived mesenchymal stem cells exhibited increased intracellular and mitochondrial ROS levels^49^. NDUFS8 was upregulated in frozen-thawed sperm of *Bos grunniens* in contrast to that in fresh sperm^50^. Therefore, we speculate that decreased intracellular ATP levels are associated with the related DEPs. Although CYB5D1, DNALI1, FABP9, and HIBADH positively correlated with cell viability, no significant reduction was observed in cell viability between each of the treatment groups compared with the control (Fig. 1B and Supplementary Table S2).

The selenoprotein phospholipid hydroperoxide glutathione peroxidase GPX4 is an antioxidant enzyme that protects cells from ferrotopsis by preventing lipid peroxidation^51,52^. Previously, GPX4 was found to be primarily localized in the mitochondria of sperm and may play a role in antioxidant activity^52^. Moreover, spermatocyte specific *Gpx4* knockout male mice showed abnormalities in spermatozoa such as mitochondrial dysfunction, decreased sperm cell count, and progressive motility, resulting in infertility^53^. Consistent with the literature, our study showed that GPX4 was related to flagellated sperm motility and fertilization (Fig. 5). These results reflect those of Ryu et al.^54^ who also reported that GPX4 was related to sperm abnormality, male and female infertility, and embryo death.

In the present study, we elucidated the alterations in sperm function and function-related protein expression in fipronil-affected spermatozoa. Briefly, fipronil diminished sperm motility, kinematics, sperm capacitation, and intracellular ATP level, and the most obvious finding of this study was that fipronil caused alteration in sperm proteins accompanied by an increase of acrosome reaction. Based on these results, we speculate that the interaction between diminished DEPs is involved in the functional and structural dysfunction of spermatozoa. These findings have significant implications for understanding how fipronil causes deficiencies in spermatozoa and how it is linked to male fertility. Therefore, these proteins may be used as novel predictive biomarkers in the estimation of sperm molecular deficiencies. Ultimately, our study demonstrated that fipronil negatively affects the expression of sperm function-related proteins as well as directly affects sperm physiological parameters such as motility and acrosome reaction. While our study provides valuable insights into the alterations in sperm function and protein expression, the exact mechanisms of fipronil on spermatozoa remain not fully understood. However, our findings confirmed that the identified DEPs are associated with specific mechanisms such as the flagellum and mitochondria. Therefore, further studies are suggested to reveal the exact mechanisms of fipronil focused on specific sperm pathways. Altogether, these findings shed new light on the molecular mechanisms of spermatozoa, contributing to an in-depth understanding of fipronil-induced sperm dysfunctions and emphasizing the potential toxicity of fipronil to male fertility.

## Methods

### Ethics statement

All animal studies were conducted in compliance with the National Research Council's ‘Guide for the Care and Use of Laboratory Animals’ and followed the ARRIVE guidelines for ethical research. This study received ethical approval from the Institutional Animal Care and Use Committee of Kyungpook National University, with approval number KNU 2019–0089.

### Chemicals and media

To induce sperm capacitation, 0.4% bovine serum albumin was added to modified Tyrode’s medium (97.84 mM NaCl, 1.42 mM KCl, 0.47 mM MgCl_2_·6H_2_O, 0.36 mM NaH_2_PO_4_·H_2_O, 5.56 mM d-glucose, 25 mM NaHCO_3_, 1.78 mM CaCl_2_.2H_2_O, 24.9 mM Na-lactate, 0.47 mM Na-pyruvate, 2 µg/mL gentamycin, and 0.005 mM phenol red). Unless otherwise stated, all chemicals and reagents were obtained from Sigma-Aldrich, St Louis, MO, USA.

### Sample collection and treatment

ICR male mice (Nara Biotech, Seoul, Korea) were maintained individually providing ad libitum feeding (Cargill Agri purina, Inc., Seongnam, Korea) and water under the conditions of 21 °C ± 2 °C and 40%–60% humidity in 12-h light/dark cycles. Epididymal sperm were collected from ICR mice at the age of 8–12 weeks. Briefly, fat-removed cauda epididymis was placed in cell culture dishes with medium and punctured using a syringe to release spermatozoa. Then, the entire collected epididymal spermatozoa were incubated with various concentrations of fipronil (0.1, 1, 10, 100, and 300 μM), including a control, at 37 °C and 5% CO_2_ for 90 min to induce capacitation. The concentration of fipronil was used according to previous studies^10,11^.

### Assessment of sperm function and kinematic analysis of spermatozoa

The computer-assisted sperm analysis (CASA) system (FSA2016, Medical supply, Seoul, Korea), an OLYMPUS BX43 phase-contrast microscope (Olympus, Tokyo, Japan), and CMOS CAMERA, 2048 × 1536 (300 M pixel), 60 Frame (Medical supply) were used to evaluate sperm motility and kinematic parameters [MOT, sperm motility (%); Progressive, progressive motility (%); VCL, curvilinear velocity (μm/s); VSL, straight-line velocity (μm/s); VAP, average path velocity (μm/s); BCF, beat cross frequency (Hz); DNM, mean dance (µm); and ALH, amplitude of lateral head displacement (µm)]. Briefly, a 10 μL sample was transferred to a preheated Makler counting chamber (Sefi Medical Instruments, Haifa, Israel) and observed. Capacitation status was evaluated by combined Hoechst33258/chlortetracycline fluorescence staining. After the induction of capacitation, 135 µL of the sample was incubated with 15 μL of H33258 solution (10 μg H33258/mL PBS) for 10 min at RT. Then, 250 µL of 2% (w/v) polyvinylpyrrolidone was added and washed. The sample was then resuspended in 100 μL of PBS and 100 μL of CTC solution (750 mM CTC in 5 μL buffer: 20 mM Tris, 130 mM NaCl, and 5 mM cysteine, pH 7.4). After incubation for 20 min at 4 °C, an OLYMPUS BX43 epifluorescence illumination using ultraviolet BP 340–380/LP 425 and BP 450–490/LP 515 excitation/emission filters (Olympus) was used to evaluate the capacitation status. Subsequently, the spermatozoa were categorized into three groups (AR, acrosome-reacted; B, capacitated; and F, non-capacitated).

### Intracellular ATP level and cell viability

Intracellular ATP and cell viability levels were measured using ATP assay kit (Abcam, Cambridge, UK) and cell cytotoxicity assay kit (Abcam), respectively, according to the manufacturers’ instructions. The absorbance of intracellular ATP and cell viability was measured using GloMax Discover (Promega, Madison, WI, USA).

### Two-dimensional electrophoresis

Two-dimensional electrophoresis was performed to elucidate differentially expressed proteins. To extract proteins, the samples were incubated in a rehydration buffer at 4 °C for 1 h (7 M urea, 2 M thiourea, 4% CHAPS (w/v), 0.05% Triton X-100, 24 μM PMSF, 1% octyl β-d-glucopyranoside, 20 mM DTT, 0.5% IPG buffer, and 0.005% bromophenol blue). Then, 250 µg of solubilized protein was placed on Immobiline DryStrip (pH 3–11 NL, 24 cm; Cytiva, Marlborough, MA, USA) for 12 h at 4 °C. First-dimension electrophoresis was performed using an IPGphor isoelectric focusing apparatus. The strips were isoelectric focused at 100 V for 1 h, 200 V for 1 h, 500 V for 1 h, 1000 V for 1 h, 5000 V for 1.5 h, 8000 V for 1.5 h, and 8000–90,000 V for 1 h. After isoelectric focusing, the strips were equilibrated with equilibration buffer A [6 M urea, 75 mM Tris–HCl (pH 8.8), 30% (v/v) glycerol, 2% (w/v) SDS, 0.002% (w/v) bromophenol blue, and 2% (w/v) DTT], and the second equilibration was performed using equilibration buffer B [6 M urea, 75 mM Tris–HCl (pH 8.8), 30% (v/v) glycerol, 2% (w/v) SDS, 0.002% (w/v) bromophenol blue, and 2.5% (w/v) iodoacetamide]. Second-dimension electrophoresis was performed using 12.5% (w/v) SDS-PAGE gels, with the strips being isoelectric focused at 100 V for 1 h and 500 V until the front of bromophenol blue began to move off the end of the gels. The gels were silver-stained according to the manufacturer’s instructions (Amersham Biosciences, Piscataway, NJ, USA), with the molecular weights ranging from 6.5 to 200 kDa and the pH levels ranging from 3 to 11. GS-800 calibrated Imaging Densitometer (Bio-Rad) was used to scan the gel for comparison and identification of the spots between each group. Then, the numerical value of protein expression level was computed using the PDQuest 8.0 software (Bio-Rad).

### In-gel digestion

Proteins were subjected to in-gel trypsin digestion. Under shaking for 5 min, the excised gel spots were destained with 100 μL of destain solution (30 mM potassium ferricyanide, 100 mM sodium thiosulfate), and the solution was removed. Then, the gel spots were incubated with 200 mM ammonium bicarbonate for 20 min. The gel fragments were dried in a vacuum centrifuge after dehydration with 100 μL of acetonitrile. This procedure was repeated three times. The dried gel fragments were then rehydrated for 45 min on ice with 20 μL of 50 mM ammonium bicarbonate containing 0.2 μg of modified trypsin (Promega). After removing the solution, 70 μL of 50 mM ammonium bicarbonate was added. Protein digestion was performed overnight at 37 °C. Finally, a nano C18 column was used to desalinate the peptide solution.

### Desalting and concentration

Custom-made chromatographic columns were used for desalting and concentration of the peptide mixture before mass spectrometric analysis. A column consisting of 100–300 nL of Poros reverse-phase R2 material (20–30 μm bead size, PerSeptive Biosystems) was packed in a constricted GELoader tip (Eppendorf, Hamburg, Germany). A 10-mL syringe was used to force the liquid through the column by applying gentle air pressure. Next, 30 μL of the peptide mixture from the digested supernatant was diluted in 30 μL of 5% formic acid, loaded onto the column, and washed with 30 μL of 5% formic acid. For analyses by tandem mass spectrometry (MS/MS), the peptides were eluted with 1.5 μL of 50% methanol/49% H_2_O/1% formic acid.

### LC–MS/MS

The samples were resuspended in 0.1% formic acid in DW for using Ultimate 3000 (Thermo Fisher Scientific, Inc.). An autosampler was used to load 2-μL aliquots of the peptide solution into a C18 column (75 μm × 15 cm, particle size 2 μm) at a flow rate of 300 nL/min. The mobile phase A contained 0.1% formic acid in DW, and the mobile phase B contained 0.1% formic acid in 90% acetonitrile. The LC gradient elution started from 5 to 95% mobile phase B within 47.5 min and held at 95% for 5.0 min. Finally, back to 5% mobile phase B for 5.0 min. The mass spectrum (MS) scan range was 150 − 2000 m/z.

### Database search

An MS/MS ion search was assigned as the ion search preference in the MASCOT software (version 2.4.1, Matrix Science, Boston, MA, USA). Peptide fragment files were obtained from the peptide peaks in ESI–MS by ESI–MS/MS. Trypsin was selected as an enzyme with a maximum of two missed cleavage sites. ESI-TRAP was selected as the instrument type. The peptide fragments were searched based on the database using the MASCOT software (version 2.4.1, Matrix Science) and FASTA search engine, and the search was limited to *Mus musculus* taxonomy in NCBInr and UniprotKB/swissprot databases. The mass tolerance was set at ± 10 ppm and ± 0.8 Da for the peptides and fragments, respectively. High scoring was defined as those greater than the default significance threshold in MASCOT (*p* < 0.05, peptide score, > 55).

### Western blot analysis

The differentially expressed proteins were validated by western blotting analysis. Briefly, each group of samples were lysed using modified Laemmli sample buffer (315 mM Tris, 10% glycerol, 10% sodium dodecyl sulfate, 5% 2-mercaptoethanol, and 5% bromophenol blue). Then, the lysates were segregated by 12% SDS-PAGE (Mini PROTEIN Tetra Cell, Bio-Rad) and transferred to Immun-Blot polyvinylidene difluoride membranes (Bio-Rad). For blocking, the membranes were incubated with 3% ECL blocking agent (GE Healthcare, Chicago, IL, USA) in DPBS at RT for more than 2 h. Then, the membranes were washed with DPBS containing 0.01% Tween-20 (PBS-T) and incubated with primary antibodies diluted in 3% ECL blocking agent (GE Healthcare) [C6orf206 polyclonal antibody (1:300; Invitrogen, Thermo Fisher Scientific, Inc., Waltham, MA, USA), CYB5D1 polyclonal antibody (1:500; MyBioSource, San Diego, CA, USA), DNALI1 polyclonal antibody (1:4,000; MyBioSource), FABP9 monoclonal antibody (1:1000; Invitrogen), HIBADH antibody (1:500; MyBioSource), NDUFS8 polyclonal antibody (1:3000; Invitrogen)]. Anti-⍺-tubulin mouse antibody (1:5000; Abcam) was used as a loading control. After treatment, the membranes were washed with PBS-T. Anti-rabbit IgG, HRP-linked antibody (1:2000; Cell Signaling Technology, Danvers, MA, USA), or goat anti-mouse IgG H&L (HRP) (1:2000; Abcam) secondary antibody was diluted in 3% ECL blocking agent (GE Healthcare) and added. Protein bands were visualized using the iBright CL1500 imaging system (Invitrogen) with an ECL Substrate (Bio-Rad). Finally, the protein expression signals from each gel were measured using the Image Lab software, version 6.1.0 (Bio-Rad), and calculated relative to the control.

### Functional annotation, protein–protein interaction (PPI) network, and signaling pathway

The biological process (BP), cellular component (CC), molecular function (MF), and pathway properties of DEPs were annotated using Gene Ontology (GO) and Kyoto Encyclopedia of Genes and Genomes (KEGG) databases by Database for Annotation, Visualization, and Integrated Discovery 6.8 (DAVID) (https://david.ncifcrf.gov/home.jsp). The PPI network was constructed using the STRING applications (version 11.5) of Cytoscape (version 3.9.1)^55^. To visualize a signaling pathway, Pathway Studio (Version 12.4.0.5, Elsevier, Amsterdam, Netherlands) was implemented.

### Statistical analysis

Data management and analysis were performed by one-way ANOVA and Pearson correlation using SPSS (Version 26.0, IBM, Armonk, NY, USA). Numerical data are represented as mean ± SEM. The level of significance was set at *p* < 0.05.

### Supplementary Information

Supplementary Information.

## Data Availability

All the data generated or analysed during this study are included in this published article [and its supplementary information files].
