# Supplementary material for: Proteomic analysis of fipronil-induced molecular defects in spermatozoa
Source: Sci Rep. 2024 Apr 1;14:7668. doi: 10.1038/s41598-024-57876-4 (PMC10985004; doi:10.1038/s41598-024-57876-4)
Supplement: Supplementary file 1 — Supplementary Information. [file 41598_2024_57876_MOESM1_ESM.pdf]

Supplementary Information

Proteomic Analysis of Fipronil-Induced Molecular Defects in Spermatozoa

Jeong-Won Bae[a] and Woo-Sung Kwon[a,b,*]

[a]Department of Animal Science and Biotechnology, Kyungpook National University, Sangju, Gyeongsangbuk-do 37224, Republic of Korea

[b]Research Institute for Innovative Animal Science, Kyungpook National University, Sangju, Gyeongsangbuk-do 37224, Republic of Korea

[*]Corresponding author:

Department of Animal Science and Biotechnology, Kyungpook National University

Research Institute for Innovative Animal Science, Kyungpook National University

Tel: +82.54.530.1942

Fax: +82.504.242.9851

E-mail: wskwon@knu.ac.kr

ORCID: 0000-0002-0848-7189

Supplementary Table S1. Matched sequence and coverage of identified differentially expressed (> 3-fold) proteins.

Accession	Symbol	Sequence	Coverage (%)
Q8K3J1	NDUFS8	K.SATDNAAR.I<br>R.YPSGEER.C<br>R.GEHALRR.Y<br>R.YDIDMTK.C<br>R.YDIDMTK.C + Oxidation (M)<br>R.FRGEHALR.R<br>R.RYPSGEER.C<br>K.EQESEVDMK.S<br>K.EQESEVDMK.S + Oxidation (M)<br>R.FRGEHALRR.Y<br>R.ILMWTELIR.G<br>R.ILMWTELIR.G + Oxidation (M)<br>K.KEQESEVDMK.S<br>K.KEQESEVDMK.S + Oxidation (M)<br>R.TTRYDIDMTK.C<br>R.TTRYDIDMTK.C + Oxidation (M)<br>R.EPATINYPFEK.G<br>R.RTTRYDIDMTK.C<br>R.RTTRYDIDMTK.C + Oxidation (M)<br>R.YPSGEERCIACK.L<br>R.RYPSGEERCIACK.L<br>R.GEHALRRYPSGEER.C<br>K.YVNKKEQESEVDMK.S<br>K.YVNKKEQESEVDMK.S + Oxidation (M)<br>K.EQESEVDMKSATDNAAR.I<br>K.EQESEVDMKSATDNAAR.I + Oxidation (M)<br>K.KEQESEVDMKSATDNAAR.I<br>K.KEQESEVDMKSATDNAAR.I + Oxidation (M)<br>K.LCEAICPAQAITIEAEPR.A	43.4
Q64433	HSPE1	R.DSDILGK.Y<br>R.SAAETVTK.G<br>K.GGIMLPEK.S<br>K.DYFLFR.D<br>K.GGIMLPEK.S + Oxidation (M)<br>K.FLPLFDR.V<br>K.SGEIEPVSVK.V<br>K.VLLPEYGGTK.V<br>R.DSDILGKYVD.-<br>K.GGIMLPEKSQGK.V<br>K.GGIMLPEKSQGK.V + Oxidation (M)<br>K.VLQATVVAVGSGGK.G<br>R.VLVERSAAETVTK.G<br>K.SGEIEPVSVKVGDK.V<br>K.VLQATVVAVGSGGKGK.S<br>K.VGDKVLLPEYGGTK.V<br>R.SAAETVTKGGIMLPEK.S + Oxidation (M)	86.3

		R.SAAETVTKGGIMLPEKSQGK.V + Oxidation (M)	
O70325-3	GPX4	R.QRGGSPR.G<br>K.DLPCYL.-<br>K.FDMYSK.I<br>R.GMLGNAIK.W<br>K.FDMYSK.I + Oxidation (M)<br>R.GMLGNAIK.W + Oxidation (M)<br>R.YAECGLR.I<br>K.WMKVQPK.G + Oxidation (M)<br>R.SMHEFSAK.D<br>R.SMHEFSAK.D + Oxidation (M)<br>R.DDWRCAR.S<br>K.EFAAGYNVK.F<br>K.GRGMLGNAIK.W<br>K.GRGMLGNAIK.W + Oxidation (M)<br>R.QEPGSNQEIK.E<br>K.FLIDKNGCVVK.R<br>K.DIDGHMVCLDK.Y + Oxidation (M)<br>R.ILAFPCNQFGR.Q<br>R.YGPMEEPQVIEK.D<br>R.YGPMEEPQVIEK.D + Oxidation (M)<br>K.FLIDKNGCVVKR.Y<br>K.ICVNGDDAHPLWK.W<br>K.RYGPMEEPQVIEK.D<br>K.RYGPMEEPQVIEK.D + Oxidation (M)<br>K.DIDGHMVCLDKYR.G + Oxidation (M)<br>K.TDVNYTQLVDLHAR.Y	60.1
Q8C0M9	ASRGL1	R.TRDLPC.-<br>K.TGDWVAK.W<br>K.GLGGLILVNK.T<br>R.LALFHVEQGK.T<br>K.LKGLGGLILVNK.T<br>K.LQAGIDLCETR.T<br>K.WTSASMPWAAVK.N + Oxidation (M)<br>R.GNLAYATSTGGIVNK.M<br>K.LQAGIDLCETRTR.D<br>K.NGKLQAGIDLCETR.T<br>K.TVEEAAQLALDYMKSK.L<br>R.GNLAYATSTGGIVNKMVGR.V + Oxidation (M)<br>K.LQAGIDLCETRTRDLPC.-	29.4
Q6P8Y0	CFAP161	K.IVIYHR.H<br>K.GELLIQR.N<br>R.ALEQAMGINT.-<br>R.ALEQAMGINT.- + Oxidation (M)<br>R.EKGELLIQR.N<br>R.LEYEGFPVR.A<br>K.MLYLTSDHR.T + Oxidation (M)<br>K.REKGELLIQR.N	35.0

		R.ANEKIVIYHR.H<br>K.REKGELLIQR.N<br>K.NQWMLVTGNPR.N<br>K.NQWMLVTGNPR.N + Oxidation (M)<br>R.GDLSLCMSPDEVK.A<br>R.GDLSLCMSPDEVK.A + Oxidation (M)<br>K.VIIVNPDQVLGEEAGK.F<br>K.SNTMLDISKPITEDTR.A<br>K.SNTMLDISKPITEDTR.A + Oxidation (M)<br>R.NKSNTMLDISKPITEDTR.A<br>R.NKSNTMLDISKPITEDTR.A + Oxidation (M)	
Q9D0M3	CYC1	R.GFQVYK.Q<br>K.DVATFLR.W<br>K.LAYRPPK.-<br>R.SRQLPLR.T<br>R.RGFQVYK.Q<br>R.HKWSVLK.S<br>R.KLAYRPPK.-<br>R.GLLSSLDHTSIR.R<br>R.GLLSSLDHTSIRR.G<br>R.HLVGVCYTEEEAK.A<br>R.AANNGALPPDLSYIVR.A<br>K.LSDYFPKPYPNPEAAR.A	28.6
Q5NCY3	CYB5D1	R.LSARTR.N<br>K.GANYQVGR.L<br>R.FVHIPPPLPR.S<br>K.HIDPLTGCMR.Y<br>K.HIDPLTGCMR.Y + Oxidation (M)<br>K.GANYQVGRLSAR.T<br>R.KHIDPLTGCMR.Y<br>R.KHIDPLTGCMR.Y + Oxidation (M)<br>R.GLVAGPDLDNFQR.R<br>R.DIRKHIDPLTGCMR.Y<br>R.DIRKHIDPLTGCMR.Y + Oxidation (M)	22.4
Q99L13	HIBADH	K.ILNMSSGR.C<br>K.ILNMSSGR.C + Oxidation (M)<br>R.SGLDPKLLAK.I<br>K.DLGLAQDSATSTK.T<br>K.EAGEQVASSPAEVAEK.A<br>K.GSLLIDSSTIDPSVSK.E<br>K.MGAVFMDAPVSGGVGAAR.S + Oxidation (M)<br>K.MGAVFMDAPVSGGVGAAR.S + 2 Oxidation (M)<br>K.TPVGFIGLGNMGNPMAK.N + 2 Oxidation (M)<br>K.EAGEQVASSPAEVAEKADR.I	30.1
Q8BVN8	DNALI1	R.ETGICPVRR.E<br>R.EVTINCAER.G<br>K.ITELETEKR.D<br>K.AQLEGIIAPKK.-<br>R.KITELETEKR.D	33.3

		R.DLERQVNEQK.A<br>K.LPSTSCVPDPTK.Q<br>K.KHNEEIQFLKR.T<br>K.ALQAEQGKSDMER.K<br>K.ALQAEQGKSDMER.K + Oxidation (M)<br>R.KALQAEQGKSDMER.K + Oxidation (M)	
P52503	NDUFS6	R.VRFVDR.Q<br>K.VYINLDK.E<br>K.VYINLDKETK.T<br>K.TGTCGYCGLQFK.Q<br>R.IIACDGGGGALGHPK.V<br>K.ITHTGQVYDEKDYR.R<br>K.ITHTGQVYDEKDYRR.V	50.0
Q9D9V4	RSPH9	R.FARVLFWGR.I<br>R.FMGDPSHEYEHTELQK.V + Oxidation (M)<br>K.VFDEEVVVQIKEETR.L<br>R.LVSIIDQIDKAVAIIPR.G<br>R.GALFKTPFGVTHVNR.T<br>K.TPFGVTHVNR.T<br>R.TFEGLPLSEVR.K<br>R.TFEGLPLSEVRK.L<br>R.KLSSYFHFR.E<br>K.LSSYFHFR.E<br>K.SDLEPSLDFLDSLEYDIPR.G<br>R.GSWSIQMER.G<br>R.GSWSIQMER.G + Oxidation (M)<br>R.SLLWPGLTFYHAPR.T<br>R.TKNYGYIYVGTGEK.N<br>K.NYGYIYVGTGEK.N	53.0
P48678	LMNA	-.METPSQRR.A<br>-.METPSQRR.A + Oxidation (M)<br>R.SGAQASSTPLSPTR.I<br>R.LQEKEDLQELNDR.L<br>R.LRITESEEVVSR.E<br>R.ITESEEVVSREVSGIK.A<br>K.AAYEAELGDAR.K<br>R.ARLQLELSK.V<br>K.KEGDLLAAQAR.L<br>K.EGDLLAAQAR.L<br>K.EAALSTALSEKR.T<br>K.RTLEGELHDLR.G<br>K.QLQDEMLRR.V<br>R.LADALQELR.A<br>R.LADALQELRAQHEDQVEQYK.K<br>R.AQHEDQVEQYK.K<br>K.ELEKTYSAK.L<br>K.TYSAKLDNAR.Q	46.0

		K.LDNARQSAER.N	
		R.IRIDSLSAQLSQLQK.Q	
		K.LRDLEDSLAR.E	
		K.EREMAEMR.A	
		K.EREMAEMR.A + 2 Oxidation (M)	
		R.EMAEMRAR.M + Oxidation (M)	
		R.EMAEMRAR.M + 2 Oxidation (M)	
		K.LALDMEIHAYR.K	
		K.LALDMEIHAYR.K + Oxidation (M)	
		K.LALDMEIHAYRK.L + Oxidation (M)	
		K.LLEGEEERLR.L	
		R.LSPSPTSQRSR.G	
		R.GRASSHSSQSQGGGSVTK.K	
		K.LESSESRSSFSQHAR.T	
		R.SSFSQHARTSGR.V	
		R.NKSNEDQSMGNWQIR.R	
		R.QNGDDPLMTYRFPPK.F + Oxidation (M)	
		R.TVLCGTCGQPADK.A	
		R.SQSSQNCSIM.-	
		K.SQDSVLDPAER.A	
		K.WVNKHLMK.V	
		K.WVNKHLMK.V + Oxidation (M)	
		R.YRPDLVDMER.V	
		R.VDSLIPWIRQHTILMSDK.S	
		K.SFPQNPVELK.A	
		K.LIVEMLEREK.S	
		K.IQNGALNCEEK.L	
		K.YYQLEELAFR.V	
		R.LECTNLYR.K	
		R.TELVSISSSEDEGNLR.F	
		K.MSQNFHTSYVETLGK.L	
		K.MSQNFHTSYVETLGK.L + Oxidation (M)	
		K.LETQYCKLK.E	
Q9QXZ0	MACF1	R.MRHLQSLHK.F + Oxidation (M)	17.0
		R.ATAELIWLNGK.E	
		R.SLQDTAEVLSLENHPAK.Q	
		K.STLSVKAICDYR.Q	
		K.AICDYRQIEITICK.N	
		K.NDECVLEDNSQRTK.W	
		K.VMALWHQLHINTK.S	
		K.SLISWNYLR.K	
		K.DLDTVQTWSLEK.L	
		R.LRIEEEVEACK.A	
		K.AHFQHLMK.S	
		K.AHFQHLMK.S + Oxidation (M)	
		K.EETLAKVYISELK.N	
		K.MDHVYGLSTVYLNK.L	
		K.MDHVYGLSTVYLNK.L + Oxidation (M)	

K.MDHVYGLSTVYLNKLK.T
K.FSQQYSTIVKDYELQLMTYK.A + Oxidation (M)
K.DYELQLMTYK.A
R.TRYTALVTLTTQHVK.Y
R.YTALVTLTTQHVK.Y
K.VVEEEKQEHVEK.V
K.TSQIFLAKHGHK.L
K.GCRAVAGVIDLGTVEIFPIFR.A
K.LSLEEGLTR.N
R.SSKNLIDPNTAEK.V
K.VGLLDLMQRCIIHQESGLK.L
K.QLAGGMVSLK.S + Oxidation (M)
R.MTIDEAVTNNLVAAK.I + Oxidation (M)
K.VGFAAGKPPVSGPR.E
K.LQRPLLGSR.K
K.SEHDMNVNSLEK.E
K.LMGKLNMFR.G
K.HGLIGEDMARQLR.K + Oxidation (M)
R.VTLASALEKK.L
K.ISVEMEGQR.Q
K.ISVEMEGQR.Q + Oxidation (M)
R.QDEKASSDSK.V
K.EQGSHYETAGNLLSER.S
R.REMGGEQSVQMSR.E
K.GDMAAQITTR.Q
R.QHDAVIPAISEIR.E
R.EEMALSLPCSVVKVDGK.I + Oxidation (M)
K.GIEGVNPEPCR.A
K.DMKQSMAER.K + Oxidation (M)
K.DMKQSMAER.K + 2 Oxidation (M)
K.QQDTCHKK.L
K.VDLKDLQGDIQSHSTSFATAVK.D
K.EQYEVLQERTR.V
K.QTQALRDELQK.F
R.FQNLSCSLDER.S
K.LQQFMENK.S
K.DFTELQKTVQER.E
K.ETEGNVPPAKTFVSAK.E
K.YEKLWEVLR.E
R.ERQESLQTVFSR.M
K.MQEDLNSRWEK.A
R.LSGQSAISTQPEAVK.Q
R.SDLGQLDNEIK.E
R.TWLDEKQSQQAK.N
K.LERLQCQLQENEEFQK.N
K.DCMQKAQK.Y + Oxidation (M)
R.DEKAGLNQNMDAITEELQAK.T + Oxidation (M)
K.TSSLEEMTQRLK.E + Oxidation (M)
K.EFQESFKNIEK.K

		R.VSSSCLTMENK.L	
		R.EMFSQLADLDDELDGMGAIGR.D + Oxidation (M)	
		R.DTDSLQSQIEDVR.L	
		K.MLEEEGTLDLLGLKR.E + Oxidation (M)	
		R.KATVDMLQAEGGR.I + Oxidation (M)	
		K.ATVDMLQAEGGR.I	
		K.LANSEPVGTQTAK.I	
		K.QTTGEEVLLIQEK.L	
		K.FHSTYEELTGWLR.E	
		K.DSMDELFSHRGEIFSTCGEEQK.A	
		K.TECLIQQYEAVSLLNSERYAR.L	
		R.QQQEEMRQLR.E + Oxidation (M)	
		K.AENMYAQIK.D + Oxidation (M)	
		K.EIQDKLDQMVFFWEDIK.A + Oxidation (M)	
		K.FWYDMAALLTTIK.D	
		K.SIDEMNNAWENLNKTWK.E	
		K.VEVYQQQIEMEK.L + Oxidation (M)	
		K.LNHQGELMLKK.A + Oxidation (M)	
		K.HLWENLGEK.I	
		K.AGSELLESSAGDDASSLR.S	
		R.LETMNQCWESVLQK.T	
		R.LMLLSRGDSGSGSK.T	
		K.EKTLLAGDTQK.L	
		R.VKALITEHQSFMEEMTR.K + Oxidation (M)	
		R.VKALITEHQSFMEEMTR.K + 2 Oxidation (M)	
		K.ALITEHQSFMEEMTR.K + Oxidation (M)	
		R.WQQVWLLALERQR.K	
		K.SRVMDFFR.R	
		R.QEFIDGILASKFPTTK.L	
		K.LEMTAVADIFDR.D	
		K.LEMTAVADIFDR.D + Oxidation (M)	
		K.RFQVEQIGENK.Y	
		R.STVMVRVGGGWMALDEFLVK.N + Oxidation (M)	
		K.FILPEGASQGMTPFR.S	
		-.MIEPFLGTWK.L	
		-.MIEPFLGTWK.L + Oxidation (M)	
		K.LISSENFENYVR.E	
O08716	FABP9	K.LISSENFENYVRELGVECEPR.K	60.0
		R.NTEISFKLGEEFEETTADNR.K	
		K.SLITFEGGSMIQVQK.W + Oxidation (M)	
		K.MVVECTMNNVVSTR.I + Oxidation (M)	

Supplementary Table S2. Correlation between sperm parameter and expression of DEPs.

	MOT	PR	VCL	VSL	VAP	BCF	DNM	ALH	AR	B	F	ATP	Cell viability	CYB5D1	DNALI1	FABP9	HIBADH	NDUSF8	RSPH9
MOT	1	.991**	.943**	.888**	.955**	.904**	-.413*	.961**	-.493*	0.349	.521*	.691**	0.395	.762**	.788**	.728**	.690**	.711**	.791**
PR		1	.944**	.892**	.964**	.909**	-.421*	.959**	-.473*	0.347	.488*	.664**	0.359	.749**	.797**	.710**	.663**	.697**	.798**
VCL			1	.895**	.960**	.966**	-0.325	.985**	-.484*	0.314	.533*	.717**	.550*	.738**	.770**	.681**	.656**	.648**	.725**
VSL				1	.972**	.927**	-.674**	.849**	-0.412	0.24	.483*	.620**	.641**	.638**	.679**	.585*	.517*	.563*	.713**
VAP					1	.957**	-.545*	.940**	-0.461	0.303	.504*	.688**	.561*	.736**	.779**	.680**	.624**	.657**	.784**
BCF						1	-.432*	.934**	-.485*	0.313	.530*	.640**	.586*	.655**	.721**	.601*	.577*	.586*	.662**
DNM							1	-0.223	-0.057	0.132	-0.037	-0.066	-0.105	-0.064	-0.156	-0.094	-0.031	-0.101	-0.276
ALH								1	-.509*	0.379	.519*	.679**	0.424	.754**	.784**	.705**	.675**	.681**	.749**
AR									1	-.885**	-.872**	-.681**	-.624**	-.751**	-.675**	-.701**	-.735**	-.659**	-.649**
B										1	.543*	.573*	0.464	.752**	.641**	.721**	.751**	.655**	.680**
F											1	.601*	.610*	.548*	.525*	0.493	.520*	0.484	0.443
ATP												1	.690**	.782**	.755**	.678**	.736**	.594**	.690**
Cell viability													1	.523*	.487*	.478*	.486*	0.423	0.445
CYB5D1														1	.917**	.954**	.926**	.929**	.940**
DNALI1															1	.923**	.916**	.908**	.943**
FABP9																1	.971**	.985**	.943**
HIBADH																	1	.943**	.895**
NDUSF8																		1	.924**
RSPH9																			1

** Correlation is significant at the 0.01 level (2-tailed).

* Correlation is significant at the 0.05 level (2-tailed).

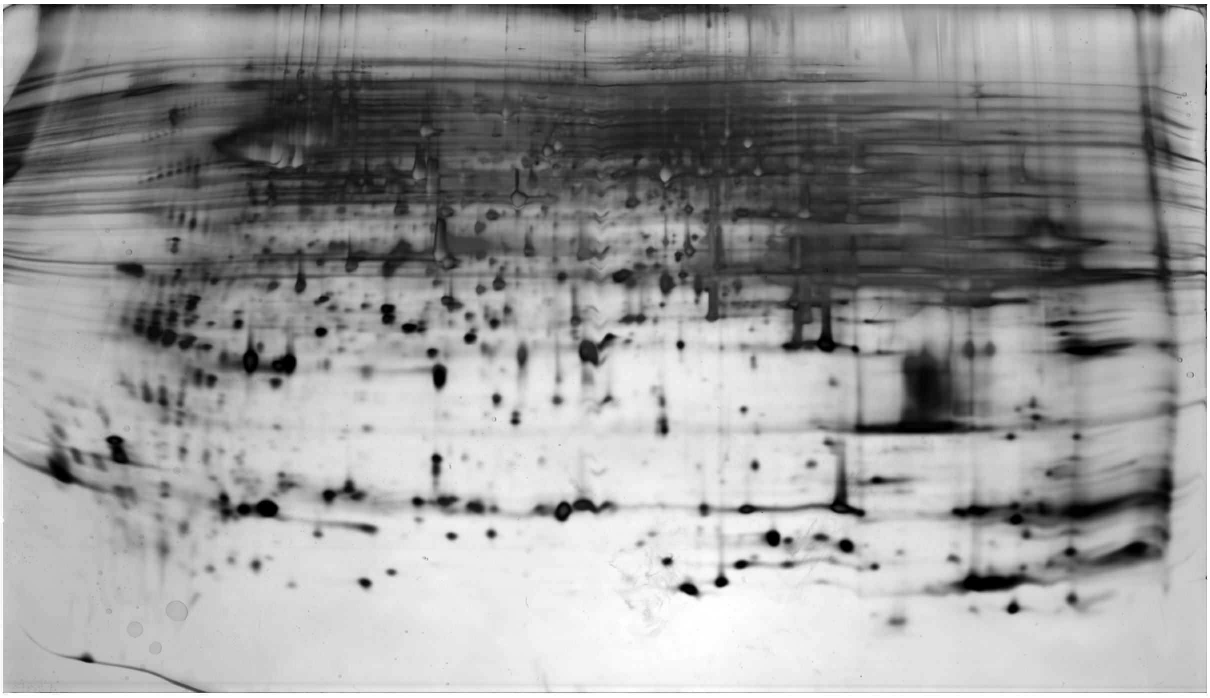

Supplementary Figure S1. Entire images of 2-DE represent control group.

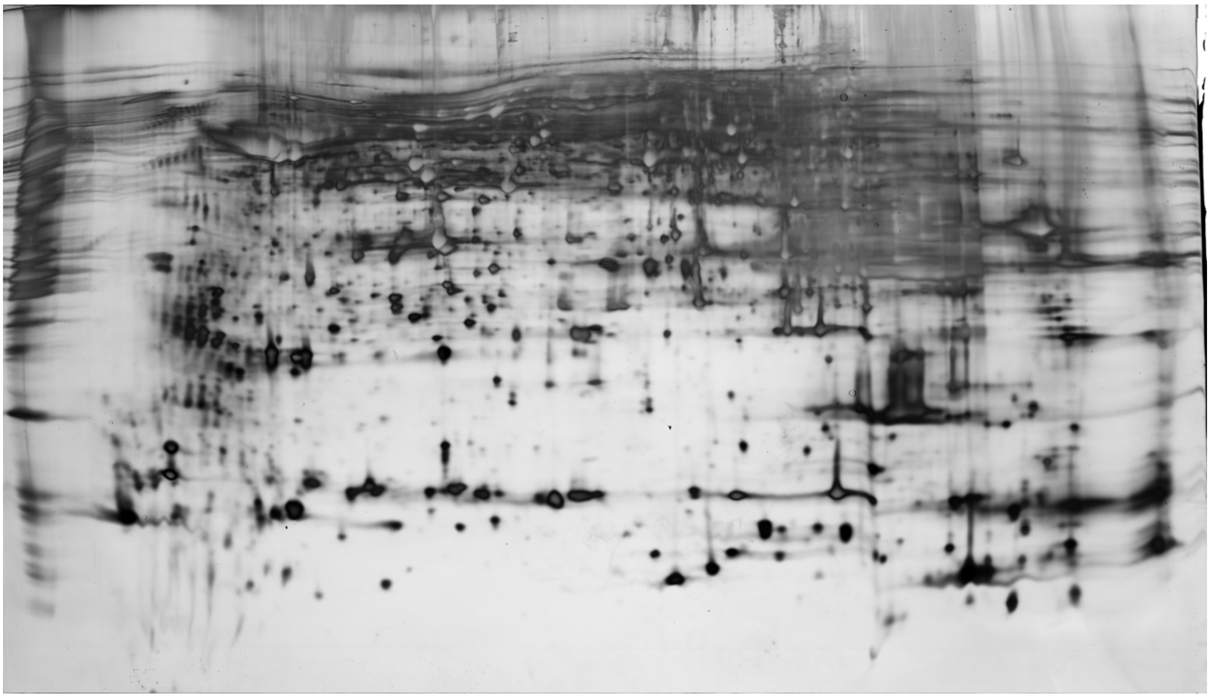

Supplementary Figure S2. Entire images of 2-DE represent 0.1 μM fipronil treatment group.

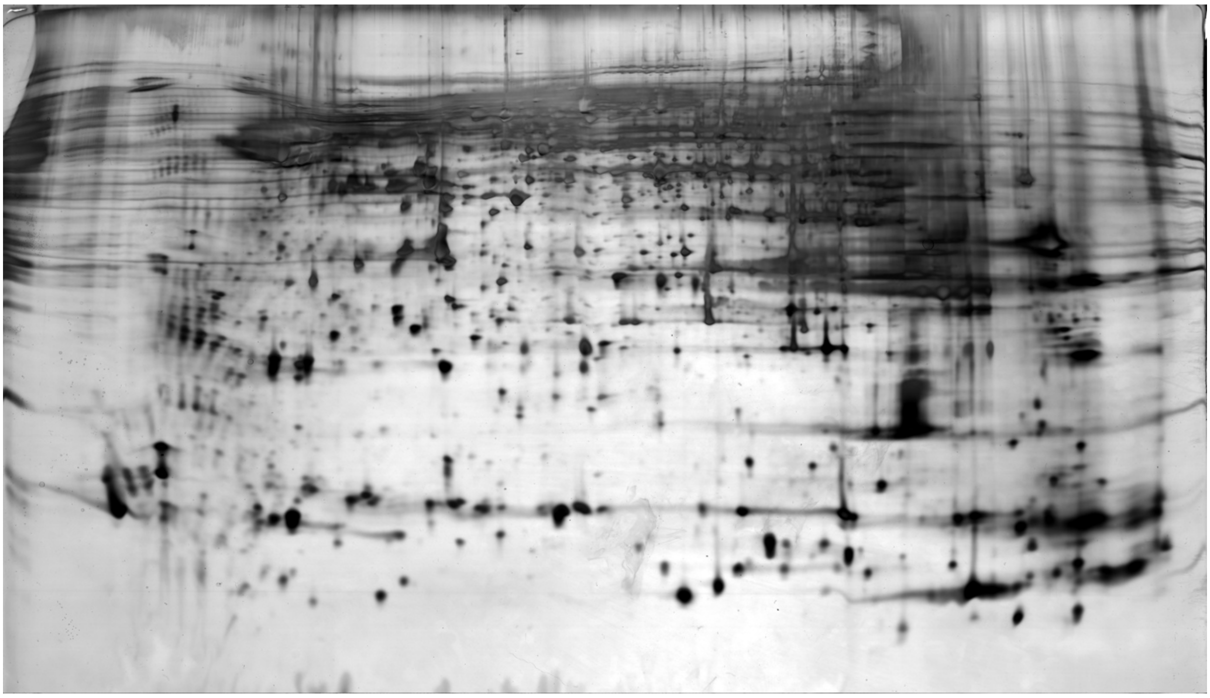

Supplementary Figure S3. Entire images of 2-DE represent 1 μM fipronil treatment group.

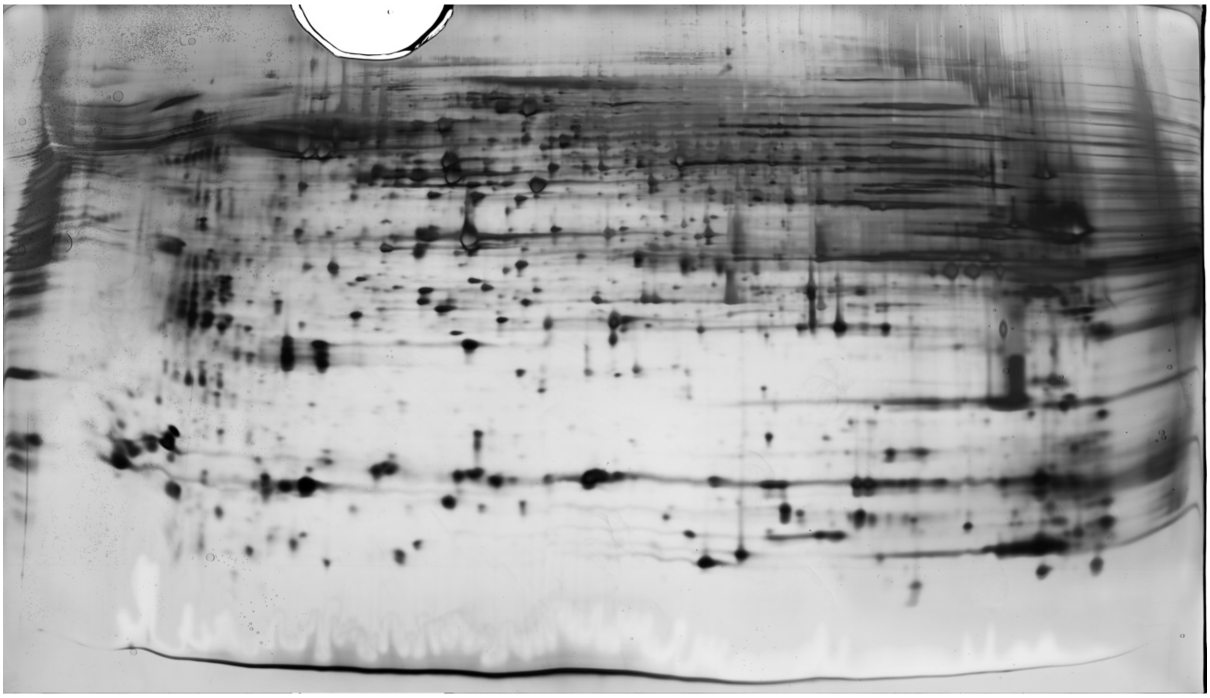

Supplementary Figure S4. Entire images of 2-DE represent 10 μM fipronil treatment group.

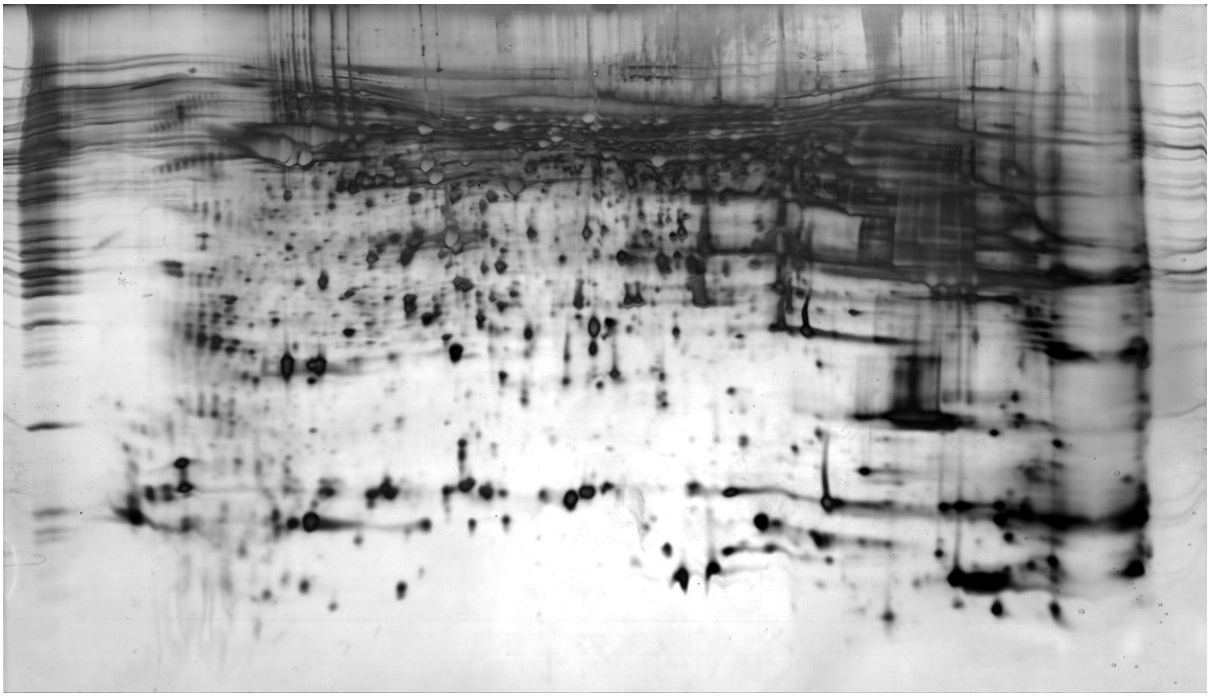

Supplementary Figure S5. Entire images of 2-DE represent 100 μM fipronil treatment group.

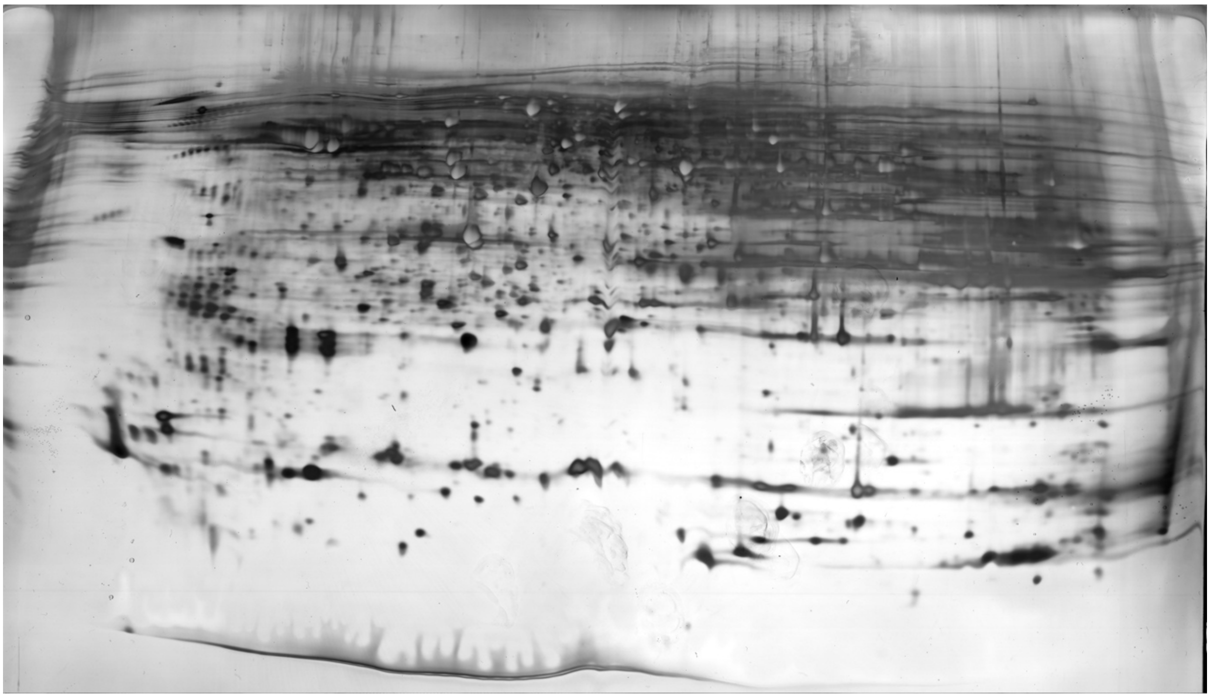

Supplementary Figure S6. Entire images of 2-DE represent 300 μM fipronil treatment group.

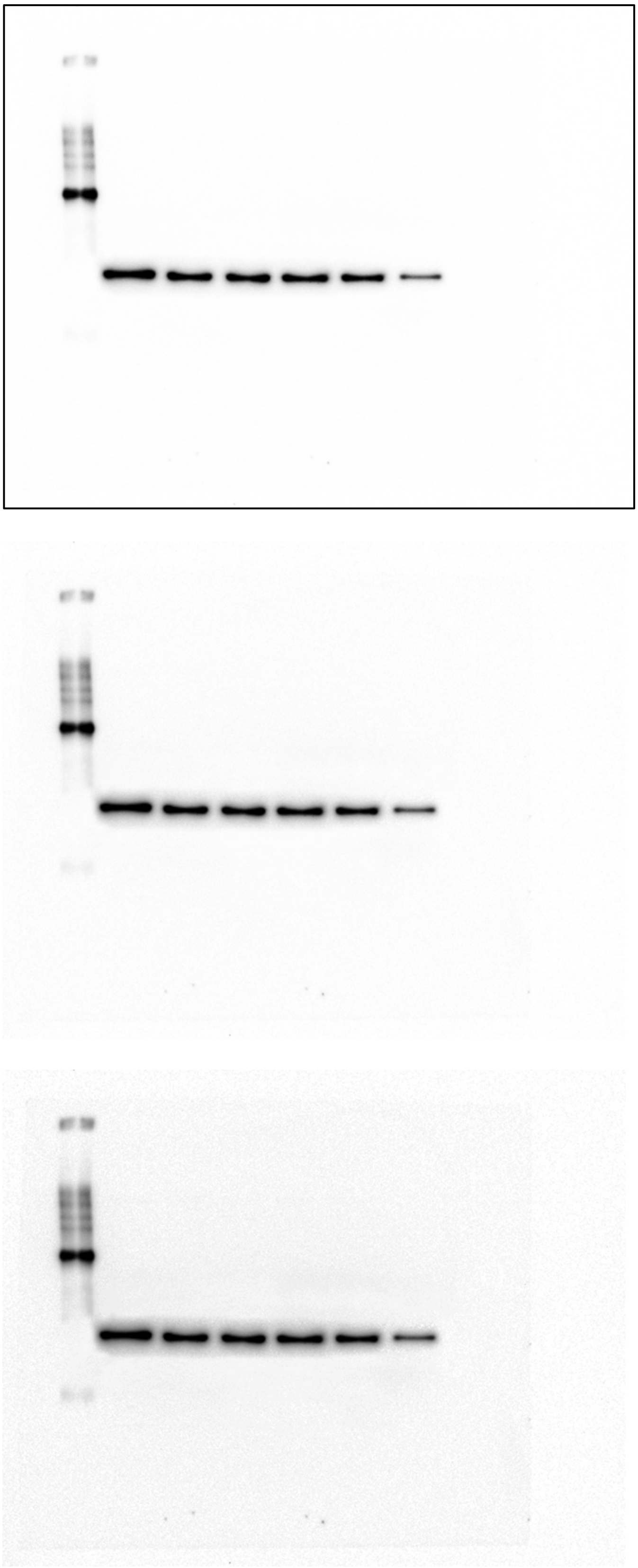

Supplementary Figure S7. Uncropped image of western blot represents NDUFS8.

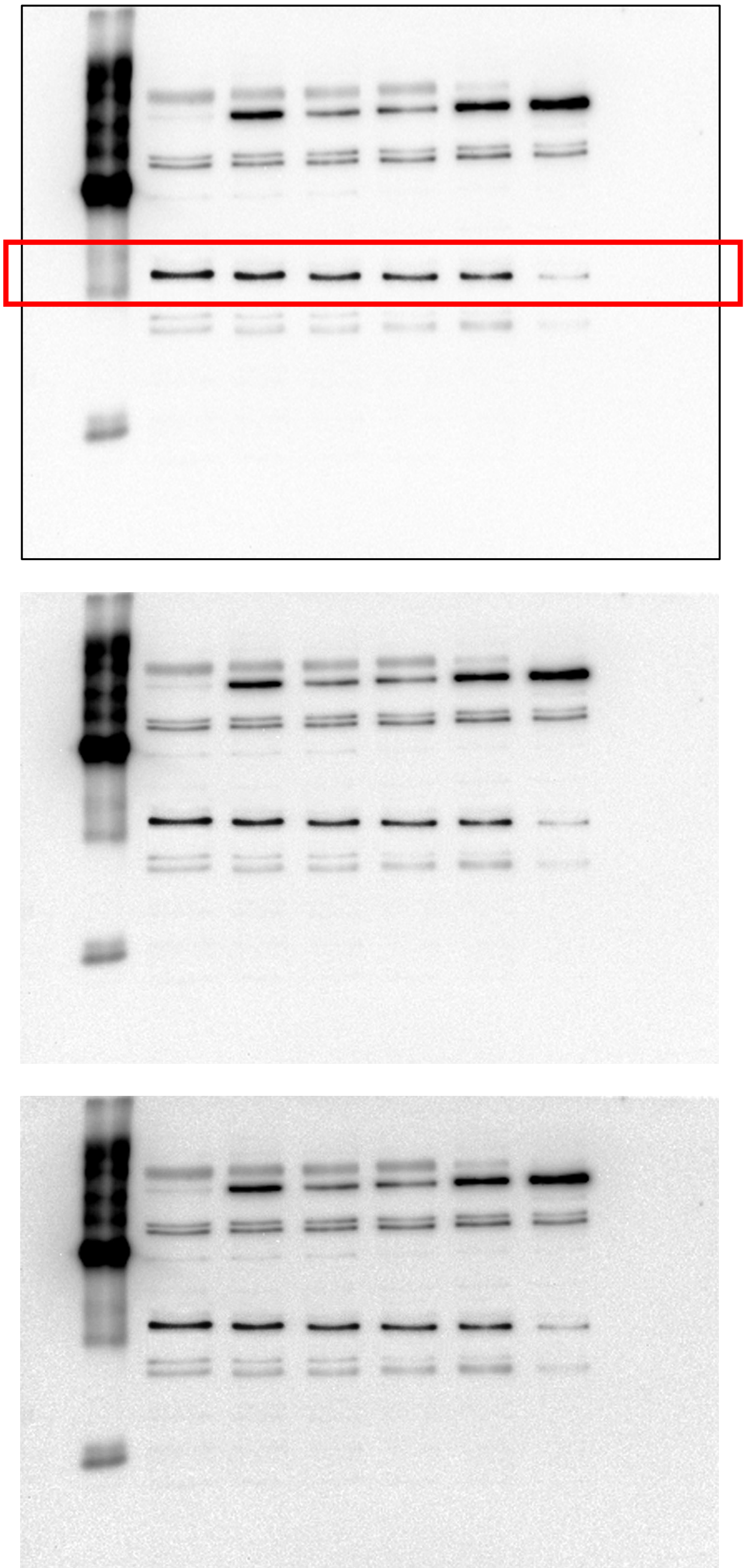

Supplementary Figure S8. Uncropped image of western blot represents CYB5D1.

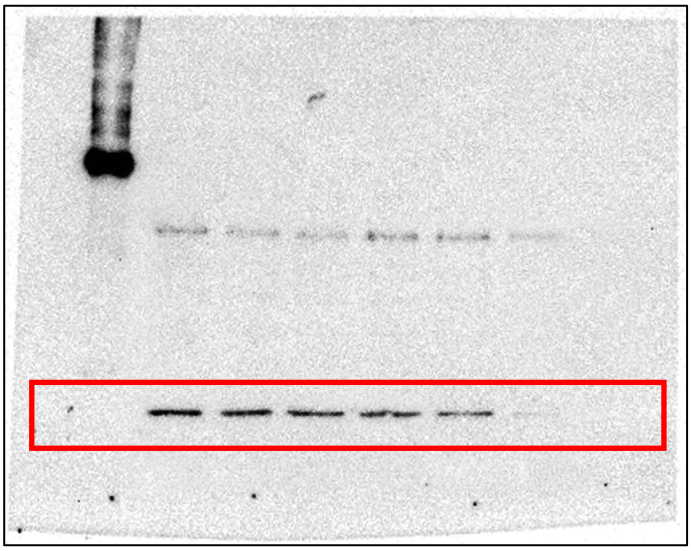

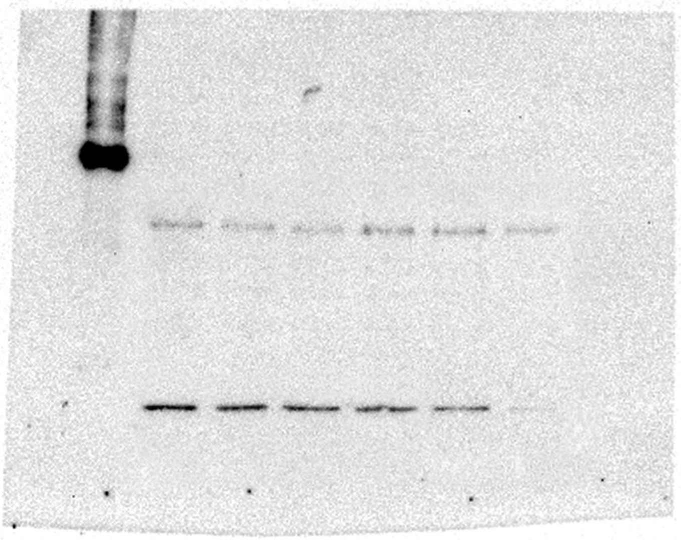

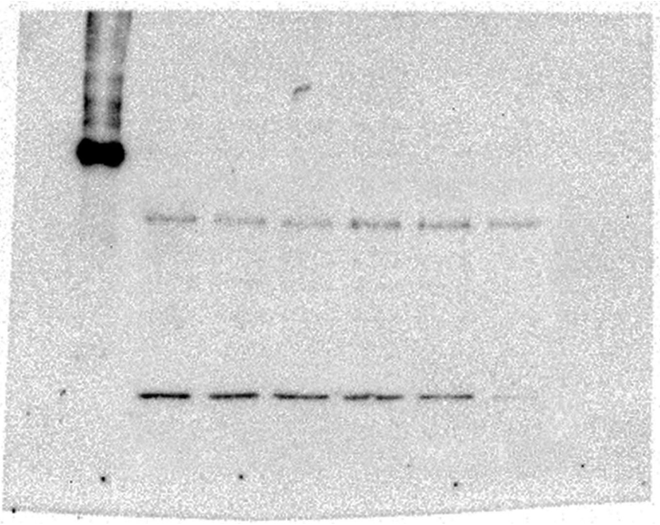

Supplementary Figure S9. Uncropped image of western blot represents HIBADH.

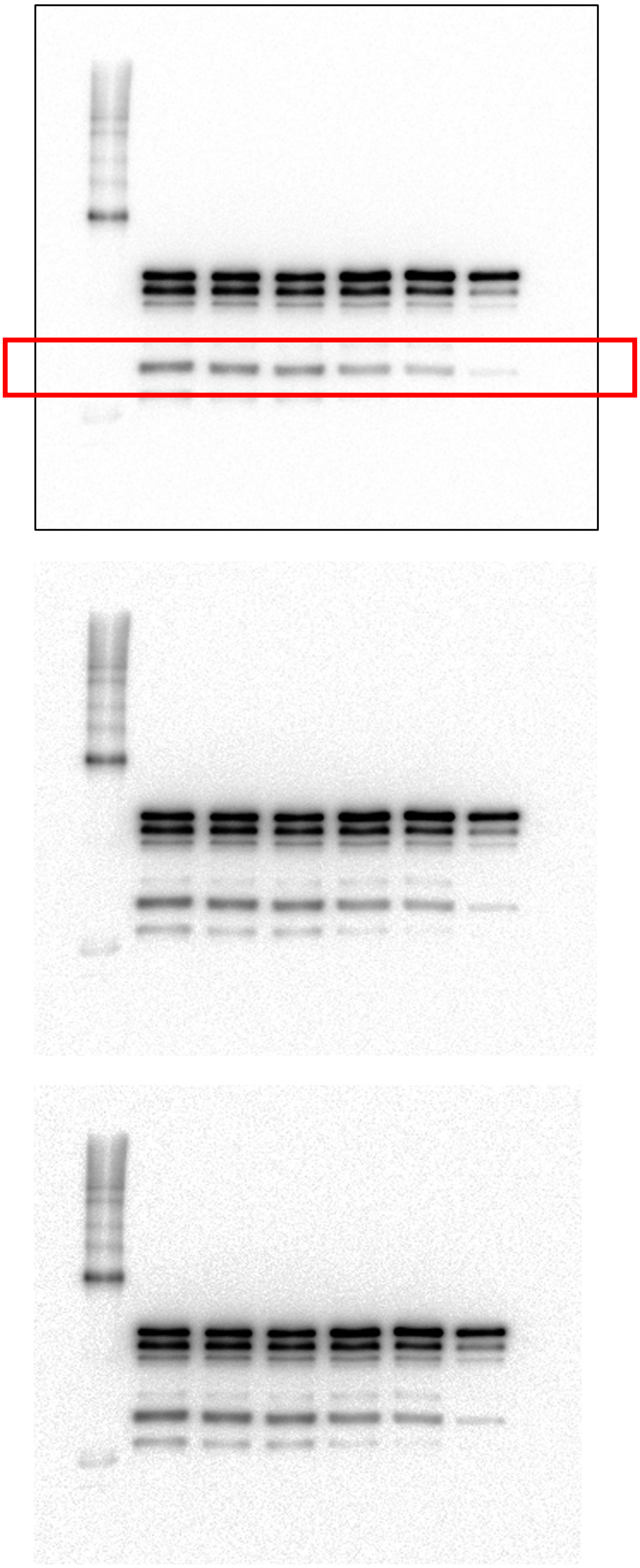

Supplementary Figure S10. Uncropped image of western blot represents DNALI1.

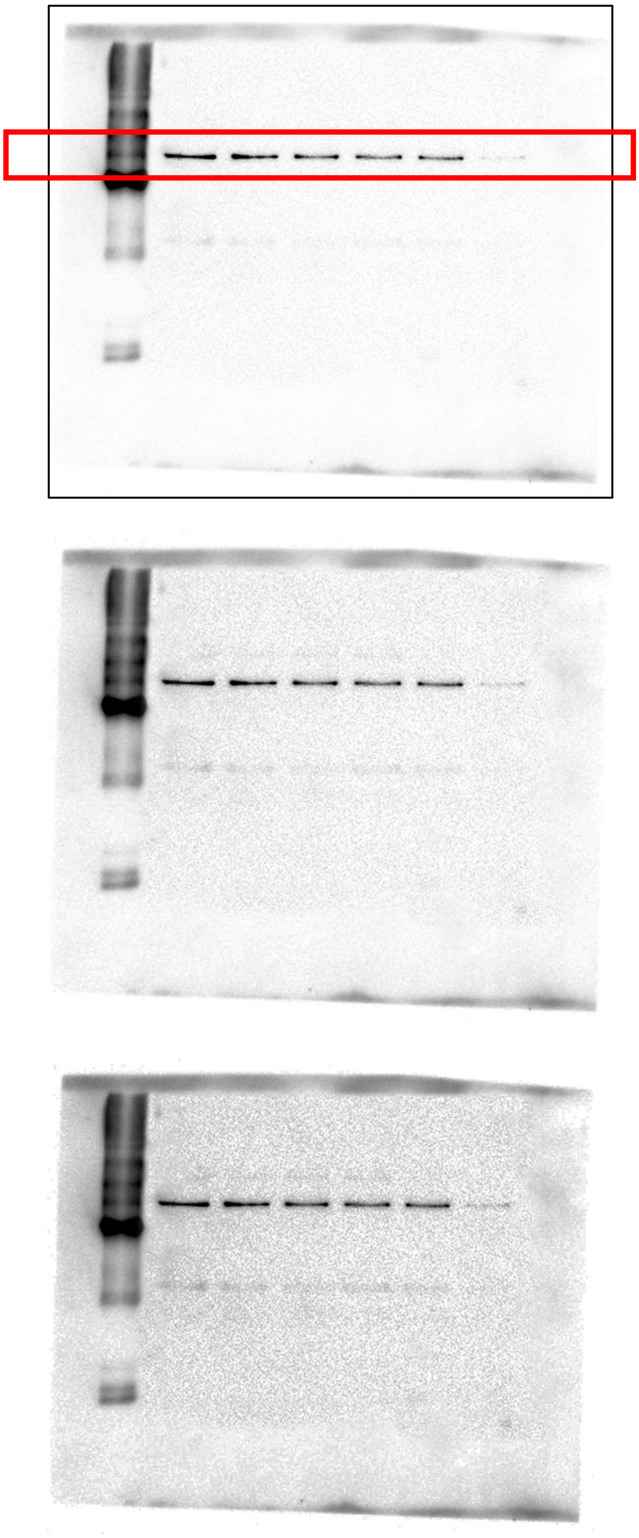

Supplementary Figure S11. Uncropped image of western blot represents RSPH9.

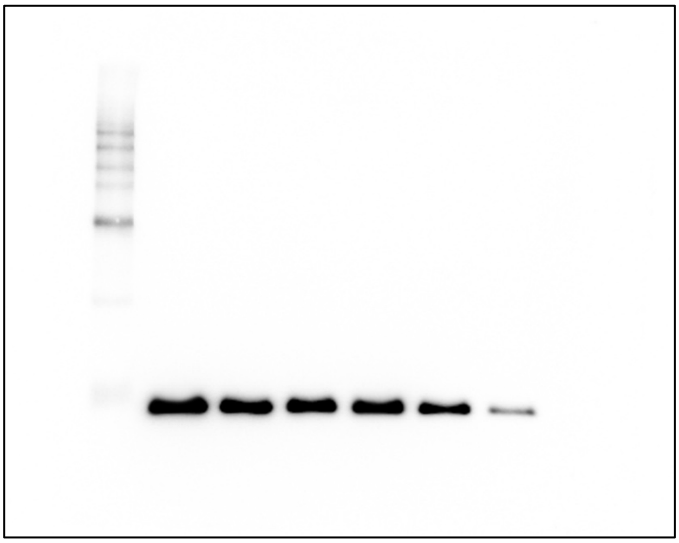

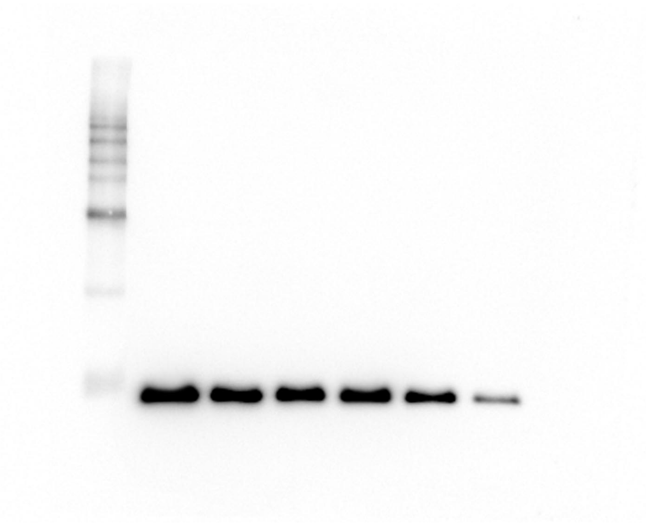

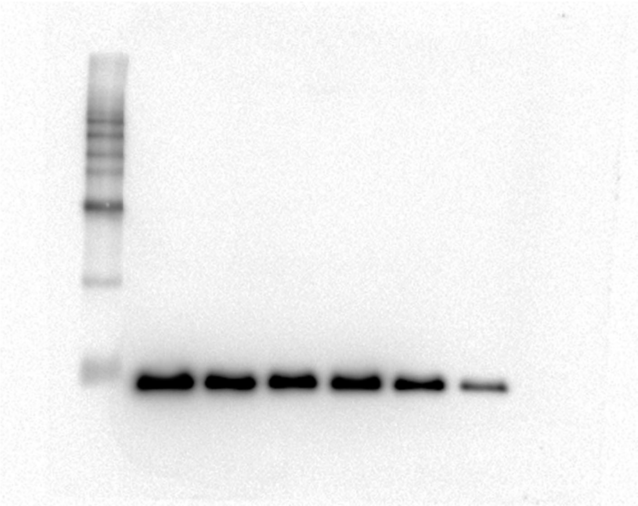

Supplementary Figure S12. Uncropped image of western blot represents FABP9.

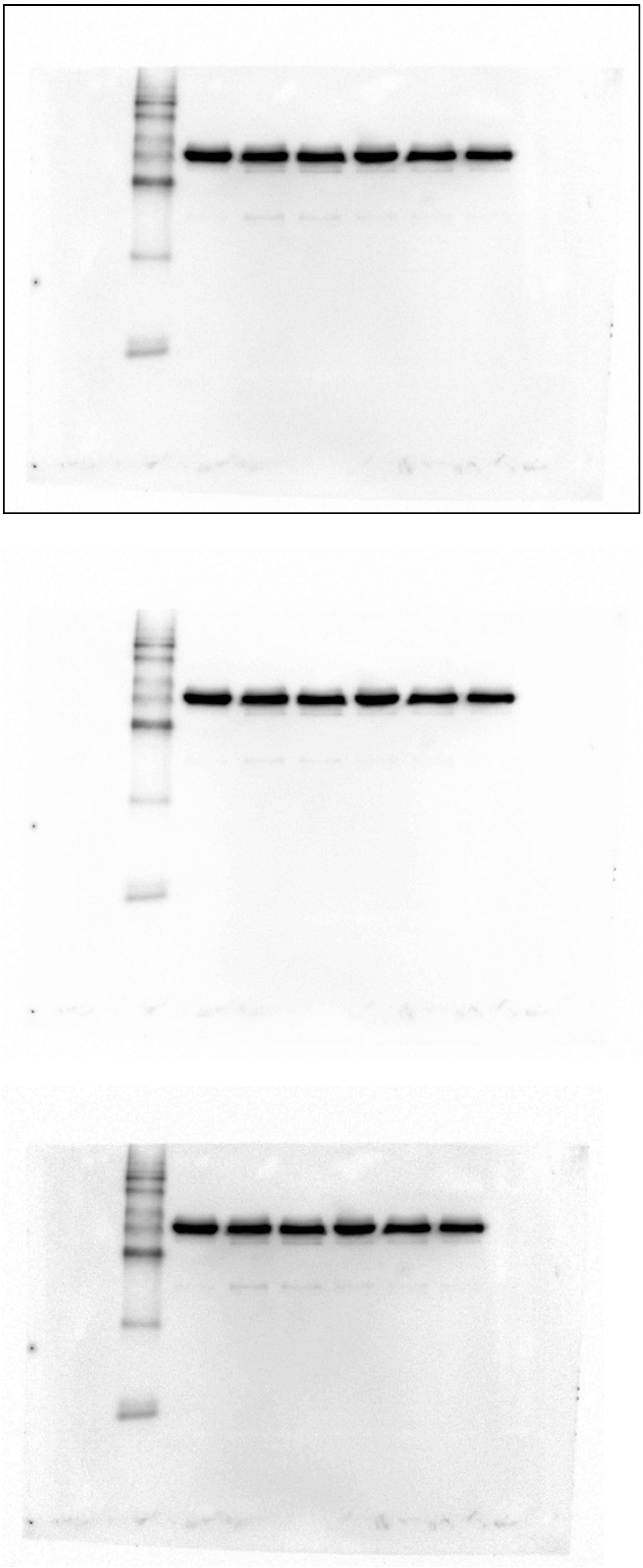

Supplementary Figure S12. Uncropped image of western blot represents tubulin.
